# Colossal infrared nonlinear optical anisotropy in a 2D charge-transfer Mott insulator

**DOI:** 10.1038/s41377-025-02130-3

**Published:** 2026-01-08

**Authors:** Ruihuan Duan, Song Zhu, Xiaodong Xu, Yao Wu, Sicheng Zhou, Xuan Mao, Zhen Xu, Wenduo Chen, Xiaodan Lyu, Youqiang Huang, Yi Zhang, Fakun Wang, Lishu Wu, Ya Deng, Manzhang Xu, Yanchao He, Jiayu Shi, Wenting Zhao, Guangtong Liu, Weibo Gao, Zhipei Sun, Xingji Li, Qi Jie Wang, Zheng Liu

**Affiliations:** 1 School of Materials Science and Engineering, Nanyang Technological University, 639798 Singapore, Singapore; 2 School of Electrical and Electronic Engineering, Nanyang Technological University, 639798 Singapore, Singapore; 3 National Key Laboratory of Microwave Photonics, Nanjing University of Aeronautics and Astronautics, Nanjing, China; 4 School of Materials Science and Engineering, Harbin Institute of Technology, Harbin, 150001 China; 5 Beijing National Laboratory of Condensed Matter Physics, Institute of Physics, Chinese Academy of Sciences, 100190 Beijing, China; 6 School of Physical and Mathematical Sciences, Nanyang Technological University, 637371 Singapore, Singapore; 7 Department of Electronics and Nanoengineering and QTF Centre of Excellence, Aalto University, Aalto, Finland; 8 Frontiers Science Center for Flexible Electronics (FSCFE) & Institute of Flexible Electronics (IFE), Northwestern Polytechnical University, Xi’an, 710072 China

**Keywords:** Optical materials and structures, Nonlinear optics

## Abstract

Mott insulators are a unique class of materials whose insulating state originates from strong electron-electron correlations: the interactions localize charge carriers, and the resulting on-site Coulomb repulsion opens a charge gap, fundamentally different from conventional insulators, making these systems an exceptional platform for exploring exotic physical phenomena. Significantly, the interplay between strong correlations and charge transfer not only stabilizes the antiferromagnetic ground state but also endows the material with enriched properties, particularly in optics. Herein, we demonstrate a 2D antiferromagnetic charge-transfer Mott insulator, Vanadium Oxychloride (VOCl), which shows giant third-harmonic generation (THG) anisotropy (*ρ*_THG_ = *I*_x_/*I*_y_, where *I*_x_ and *I*_y_ represent the THG intensities corresponding to the excitation polarization parallel to crystal’s *x*- and *y*-axes), with *ρ*_THG_ reaching up to 187 at 1280 nm excitation wavelength. Notably, it is the highest THG anisotropic ratio within the van der Waals materials family. The nonlinear anisotropy is further modulated across a broadband infrared (IR) excitation wavelength range from 2028 to 1280 nm, during which *ρ*_THG_ rises from 2.6 to 187, corresponding to a 72-fold enhancement relative to its value at 2028 nm. Additionally, VOCl demonstrates layer-independent third-order susceptibilities (χ^(3)^ ~ 10^-19^ m^2^/V^2^) and band structures attributed to its extremely weak interlayer electronic coupling. Moreover, the colossal THG anisotropic ratio in 2D VOCl can be ascribed to the synergistic effect of the correlated charge-transfer Mott insulator behavior and intrinsic *C*_3_ symmetry breaking, as supported by theoretical calculations. The colossal nonlinear optical anisotropy in 2D VOCl positions it as an excellent candidate for nanophotonic and optoelectronic applications, enabling next-generation nanodevices based on 2D correlated Mott insulators.

## Abstract (web summary)

Giant THG anisotropy in 2D VOCl emerges from a synergistic coupling of charge-transfer Mott insulator behavior with intrinsic *C*_3_ symmetry breaking.

## Graphical abstract

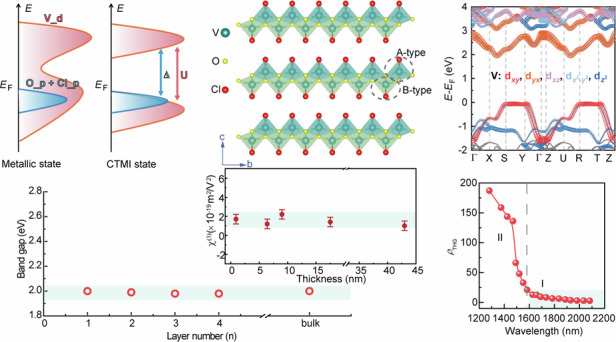

## Introduction

Nonlinear optical processes and their anisotropy are critical components in advanced optical technologies, serving as the foundation for the efficiency and capabilities of modern optics.^1–7^ In particular, two-dimensional (2D) materials’ nonlinear optics and anisotropy open a new platform for researchers to modulate and manipulate light, unlocking a broad array of applications in nanoscale photonics, such as quantum photonics, on-chip photonics, ultrafast photonic devices, data security chips, signal processors, all-optical switching, and multiplexers.^8–15^ However, as the photonic industry rapidly evolves, 2D materials with strong nonlinear responses and large optical anisotropy are urgently required to meet the increasing performance demands of nanophotonic devices.

Notably, Mott insulators exhibit superior nonlinear optical properties compared to traditional materials, making them promising candidates in the field of nonlinear optics.^16–19^ These materials feature strong electron correlations that lead to electron localization and a charge gap arising from intense on-site Coulomb interactions—distinct from conventional band insulators.^20–22^ This unique electronic structure results in an interplay across charge and spin manifolds, making the optical excited states distinctly different from those in traditional insulators.^22^ As a result, Mott insulators display a range of novel phenomena in optical and other related fields.^23^ Their enhanced nonlinear optical response primarily stems from the combined effects of electron correlation and charge transfer.^24^ Neither factor alone is sufficient to enhance nonlinear optical effects, however, when both coexist, the material’s ground state can enter an antiferromagnetic state, while the excited state undergoes charge transfer, leading to a significant enhancement in nonlinear optical effects.^24^

This study reveals that the 2D charge-transfer Mott insulator (CTMI), VOCl, shows strong nonlinear optical process, and giant linear and nonlinear optical anisotropy in the infrared regime. Because VOCl possesses inversion symmetry, electric-dipole second-harmonic generation (SHG) is forbidden,^25,26^ we therefore focus on its THG response. VOCl exhibits strong third-order susceptibility (χ^(3)^ ~ 10^-19^ m^2^/V^2^) that is independent of layer number, with its band gap (~2.0 eV) displaying a similar characteristic, which suggests that VOCl has extremely weak interlayer electronic coupling. Notably, VOCl nanoflakes exhibit colossal linear and nonlinear optical anisotropy. The anisotropic ratio for photoluminescence (PL) and THG intensities reaches up to 6.8 and 187, respectively. Moreover, as the excitation wavelength reduces from 2028 to 1280 nm, the THG anisotropic ratio increases by a factor of 72. As far as we are aware, the THG anisotropy ratio represents the highest reported to date, surpassing those observed in other van der Waals materials. Based on theoretical calculations, the enormous nonlinear optical anisotropy can be ascribed to the synergistic effect of the correlated charge transfer in the Mott insulator state and intrinsic *C*_3_ symmetry breaking. Our research underscores the promise of the 2D antiferromagnetic CTMI VOCl, with its strong nonlinear optical anisotropy, for on-chip nanophotonic devices, while simultaneously advancing the development of 2D Mott insulators in nonlinear optics.

## Results

### Crystal characterization of VOCl

**Fig. 1:**
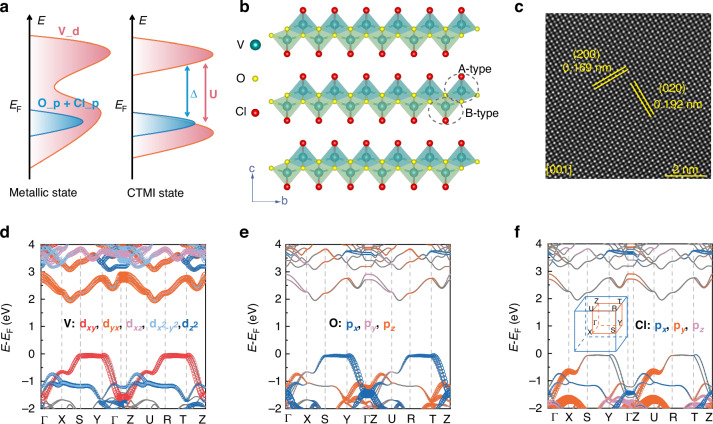
Crystal structure and synthesis of the 2D semiconductor VOCl. **a** Schematic of the charge-transfer Mott insulator state using the Hubbard U treatment, where the parameters Δ and U indicate the charge-transfer gap and on-site Coulomb interaction, respectively. **b** Structure of 2D VOCl along the *a*-axis. **c** Atomic-resolution STEM image of exfoliated VOCl flakes viewed along the [001], showing interplanar distances of the (200) and (020) planes as 0.169 nm and 0.192 nm, respectively. **d**–**f** Orbital-projected band structures for V 3 d, O 2p, and Cl 3p orbitals

As previously mentioned, Mott insulators are a class of materials with exceptional nonlinear optical properties.^17,24^ Here, density functional theory (DFT)-based simulations were conducted to investigate the 2D antiferromagnetic semiconductor VOCl. Its crystal symmetry is orthorhombic *P*mmn (No.59) which lacks *C*_3_ rotational symmetry. As shown in Fig. 1b, VOCl displays a layered structure viewed along the *c*-axis. In the asymmetric unit, a V atom binds with two Cl and four O atoms to form deformed octahedra VO_4_Cl_2_, named A-type and B-type octahedron (Figs. 1b and S1). Octahedral chains are built by the A-type (B-type) VO_4_Cl_2_ octahedrons by sharing Cl atoms along the *b*-axis, and the octahedral chains connect to each other via Cl and O atoms (shared edges in the octahedron) to form A-type (B-type) octahedral plane along the *a*-axis (Figs. 1b and S1). Finally, the monolayer VOCl is constructed by A-type and B-type octahedral planes via sharing three O atoms (a plane in the octahedron) along the *c*-axis (Figs. 1b and S1), resulting in the puckered configuration of V atoms along the (001) direction (see Fig. S1). In the DFT calculation process, the lattice of VOCl parameters were optimized to *a* = 3.35 Å, *b* = 3.77 Å, and *c* = 15.79 Å. They match the experimental results (see Figs. S2 and 1c), in which the X-ray diffraction (XRD) pattern displays *d* (001) is ~0.791 nm and the scanning transmission electron microscopy (STEM) characterization displays the interplanar distances of (200) and (020) are ~0.169 and 0.192 nm, respectively. Based on theoretical calculation, VOCl exhibits metallic behavior with the treat of semilocal functional (Fig. S3), however, experimentally, it intrinsically shows a semiconductor band gap of ~2.1 eV (Figure S4). Therefore, VOCl should be a typical correlated 2D insulator. To fully consider the strong electron-electron interaction in *d*-orbitals, an effective Hubbard on-site energy was adopted to correct the electronic structure, realizing a Mott transition, as shown in Fig. 1a. The Hubbard U term is applied to the V ions, opening a Mott-Hubbard band gap in the V 3 *d* band, with the O 2*p* and Cl 3*p* band positioned between the V 3 *d* orbitals. As shown in Fig. 1d-f, the lowest conduction band is primarily contributed by V_*d*_yz_, while V_*d*_xy_, O_*p*_x_, and Cl_*p*_y_ jointly contribute to the near *E*_F_ valence band. For the topmost valence band, V_d orbital plays the dominant role in the whole of Brillouin zone. However, Cl_*p*_y_ orbitals localize around X and U points and O_*p*_x_ orbitals mainly distribute from S to Γ point and R to Z point. Then, the charge transfer should mainly occur between V 3 *d*, O 2*p*, and Cl 3*p* orbitals, with a theoretical band gap of ~ 2.0 eV, matching the experimental observations and previous report.^27^ Thus, electronic-structure analysis identifies layered VOCl as a typical correlated CTMI (see Fig. 1a).

Inspired by the calculations, VOCl crystals with a lateral size of up to ~15 mm are successfully grown by the chemical vapor transport (CVT) approach (Fig. S2 inset). XRD pattern (Fig. S2) shows its high quality and pure phase. The X-ray photoelectron spectroscopy (XPS, Fig. S5) was adopted to quantify elemental ratios of V, O, and Cl, yielding values of 1.00:0.92:1.05. In addition, XPS spectra exhibit that V 2*p*_1/2_ and V 2*p*_3/2_ are located at 524 and 516 eV, O 1 s is positioned at 532 eV, and Cl 2*p*_3/2_ and Cl 2*p*_1/2_ are found at 199 and 200 eV, respectively. As shown in Figs. 1c and S6, the STEM images and fast Fourier-transform (FFT) pattern reveal the orthogonal symmetry of VOCl crystals and their high quality. The simulated STEM image is well fitted to the selected atomic-resolution STEM image and calculated crystal model, and all manifest the orthogonal arrangement of V atoms in the plane of (001). Moreover, the STEM EDX mapping for the exfoliated nanoflake presents the homogeneous distribution of V, O, and Cl. The magnetic characterizations for VOCl crystals are plotted in Fig. S7, which indicate the in-plane antiferromagnetic behavior for VOCl with *T*_N_ ~ 79 K.^28^

### THG process in VOCl

**Fig. 2:**
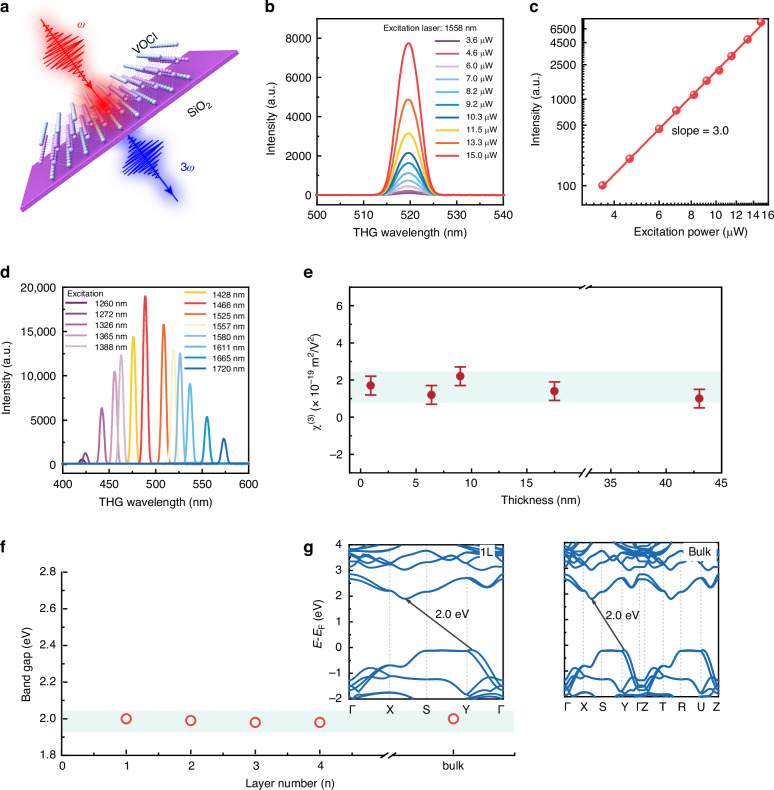
THG process in VOCl. **a** Schematic representation of the THG response in VOCl flakes. **b** THG process-dependent on excitation intensity at 1558 nm. **c** THG intensity vs. the excitation intensity. The slope of 3.0 implies the occurrence of a THG process. **d** Variation of THG intensity with excitation wavelength. **e** Variation of χ^(3)^ with thickness. **f** Band gap vs. the number of layers in VOCl flakes. **g** Calculated electronic structures for monolayer (left) and bulk (right) VOCl

Guided by the Mott insulator nature of VOCl, the THG properties were investigated using a home-built optical system under transmission configurations (see Fig. 2a and Method).^2,29^ Fig. 2b shows the THG spectra vs. pump power under 1558 nm irradiation, giving the THG emission peak at ~519 nm (*λ*/3). The log-log scaling in Fig. 2c gives a slope of ~3, confirming third-order behavior. The irradiation wavelength significantly influences the THG intensity in 2D materials. Figure 2d compares spectra of 2D VOCl acquired at identical average power for different pump wavelengths, revealing a pronounced variation with pump wavelength and a peak THG response at ~500 nm, corresponding to ~1500 nm excitation. It agrees with the absorption peak of VOCl and the theoretically calculated band gap, suggesting the strong resonance enhancement driven by interband transitions (see Figs. 2g, S4, and S8). Furthermore, monolayer VOCl exhibits a moderate third-order nonlinear susceptibility (χ^(3)^) of ~ 1.9 × 10^-19^ m^2^/V^2^ (Fig. S9), on par with BP^30^, graphene^31^, MoS_2_, MoSe_2_, WS_2_ and MoSe_2_^32^ (~ 10^-19^ m^2^/V^2^), smaller than PdPSe (6.2 × 10^-19^ m^2^/V^2^)^2^. Notably, the χ^(3)^ of VOCl remains nearly unchanged as the thickness increases, indicating the negligible interlayer coupling in VOCl (Fig. 2e). To further illustrate the suppressed interlayer interactions in VOCl, the layer-resolved electronic band structures were calculated via first-principle methods. As displayed in Figs. 2f, g, and S8, the band gap remains nearly constant (~ 2.0 eV) from the monolayer to the bulk^33^, in opposition to the behavior of other 2D materials e.g., PdSe_2_^34^, PtSe_2_^35^, PtS_2_^29^, BP^36^, where strong interlayer coupling leads to significant changes in their electronic structures and band gaps. Additionally, the layer-dependent Raman spectra show almost no shift in Raman peaks with increasing thickness (see Fig. S10), further confirming the weak interlayer electronic coupling in VOCl. This behavior is distinctly different from other 2D materials and can be attributed to VOCl’s unique structural characteristics. In VOCl’s layered structure (see Fig. 1b), the intralayer connections are primarily covalent bonds formed by V-O atoms, creating a puckered 2D framework. The Cl atoms, which encase the V-O corrugations, become inert after extracting electrons from V atoms, forming an inert outer shell that separates the layers and weakens interlayer interactions. This aligns well with the orbital-projected band structures, where the 3*p* orbitals of Cl atoms have negligible contribution to the valence band maximum (Fig. 1d-f). Furthermore, it is noteworthy that the near *E*_F_ valence bands of VOCl, spanning from the Г- to Z-points, are primarily contributed by V 3 *d*, O 2*p*, and a minor contribution from Cl 3*p* orbitals. These regions exhibit minimal dispersion, namely flat band, caused by highly localized in-plane electronic states and weak interlayer coupling in 2D VOCl. This can be attributed to its unique 2D puckered V-O configuration and the inherent characteristics of a correlated CTMI.^21,37–39^

### Colossal THG anisotropy in 2D VOCl

Motivated by electron localization and flat bands arising from strong electron correlations,^21,22^ along with *C*_3_ symmetry breaking,^40^ 2D CTMI VOCl is expected to exhibit exceptional optical anisotropy. As shown in Fig. S11, VOCl displays strong anisotropic linear optical properties. Its PL peak is located at ~2.15 eV, consistent with the experimental and theoretical band gap (Figs. S4 and 2f). The polarization-resolved PL was collected using a parallel configuration, namely, the excitation and detection polarizations are parallel, while the rotation angle *θ* varies from 0° to 360° (in the work, all *θ* = 0° means the excitation polarization direction is aligned with VOCl’s *b*-axis, see Fig. S12). As shown in Fig. S11b, angle-dependent PL intensities for exfoliated VOCl flakes exhibit two-fold symmetry, with the PL intensity reaching a maximum at *θ* = 0°. Notably, the anisotropic ratio of PL intensities is calculated using the equation of $\rho =\,\frac{{I}_{\max }}{{I}_{\min }}$, and reaches up to 6.8, surpassing that of many other 2D materials, such as GaTe (5.0)^41^, ReS_2_ (4.3)^13,42^, TiS_3_ (3.2)^43^, and GeAs (1.3)^13,44^. Figure S11c displays the angle-dependent reflectance spectra spanning 450-950 nm, revealing that VOCl flakes have two absorption peaks located at ~ 565 nm and 818 nm, and two reflectance peaks located at ~680 and 850 nm. The absorption peak position of 565 nm corresponds to 2.19 eV, slightly larger than the PL peak of ~2.15 eV. Meanwhile, over 450-950 nm, the reflectance for *b*-axis-polarized light is lower than that for *a*-axis-polarized light, which suggests that more photons are absorbed under *b*-axis-aligned excitation polarization. This is consistent with the polarization-dependent PL spectra and absorption spectra (Fig. S4a). Additionally, the polarization-dependent reflectance intensity at a wavelength of 850 nm is extracted from Fig. S11c, which shows the typical two-fold symmetry and suggests strong optical anisotropy with a ratio of ~1.4 (Fig. S11d). Furthermore, angle-resolved polarized Raman spectra (ARPRS) for 2D VOCl flakes were also collected under parallel and perpendicular geometry, respectively (Fig. S13). As shown in Fig. S13, all peaks under the parallel configuration demonstrate two-fold symmetry. These polarized linear optical properties indicate the presence of highly localized valence-band states and the breaking of *C*_3_ symmetry.

Beyond linear optical anisotropy, the anisotropy of THG was also examined, driven by the highly localized electronic CTMI states and *C*_3_ symmetry breaking in 2D VOCl.^40^ It has a *P*mmn (NO.59) space group and mmm point group, thus THG susceptibility tensor of VOCl can be expressed as^25,45^:

1 $${\chi }^{(3)}=\left[\begin{array}{cccccccccc}{\chi }_{11}^{(3)} & 0 & 0 & 0 & 0 & {\chi }_{16}^{(3)} & 0 & {\chi }_{18}^{(3)} & 0 & 0\\ 0 & {\chi }_{22}^{(3)} & 0 & {\chi }_{24}^{(3)} & 0 & 0 & 0 & 0 & {\chi }_{29}^{(3)} & 0\\ 0 & 0 & {\chi }_{33}^{(3)} & 0 & {\chi }_{35}^{(3)} & 0 & {\chi }_{37}^{(3)} & 0 & 0 & 0\end{array}\right]$$

where the first subscript (1-3) stands for the *x* (namely, *b*-axis in VOCl crystal structure), *y* (*a*-axis), and *z* (*c*-axis), respectively. The second index specifies the term defined below:

$$\begin{array}{cccccccccc}xxx & yyy & zzz & yzz & yyz & xzz & xxz & xyy & xxy & xyz\\ 1 & 2 & 3 & 4 & 5 & 6 & 7 & 8 & 9 & 0\end{array}$$

For the incident light, its electric field is given by ${{\vec{\rm{E}}}}={E}_{{x}}{\vec{\rm{x}}}+{E}_{y}{\vec{\rm{y}}}+{E}_{z}{\vec{\rm{z}}}$. Since the sample is pumped in the *x*-*y* plane in our geometry, the *z* component is neglected, thus the incident light can be further given by ${\vec{\rm{E}}}={{E}}_{0}({\vec{\rm{x}}}\,\cos (\theta )+{\vec{\rm{y}}}\,\sin (\theta ))$, where *θ* denotes the polarization angle (0° along *x*).

The χ^(3)^-induced polarization component in VOCl is expressed as:

2 $${{P}}^{(3\omega )}=\left[\begin{array}{c}{{P}}_{x}^{(3\omega )}\\ {{P}}_{y}^{(3\omega )}\\ {{P}}_{z}^{(3\omega )}\end{array}\right]={\varepsilon }_{0}{E}_{0}^{3}\left[\begin{array}{c}{\chi }_{11}^{(3)}{\cos }^{3}(\theta )+3{\chi }_{18}^{(3)}\cos (\theta ){\sin }^{2}(\theta )\\ {\chi }_{22}^{(3)}{\sin }^{3}(\theta )+3{\chi }_{29}^{(3)}\sin (\theta ){\cos }^{2}(\theta )\\ 0\end{array}\right]$$

Thus, the THG intensity resolved into *x*- and *y*-polarized components is given by:

3 $${I}_{x}^{(3\omega )}\propto ({\chi }_{11}^{(3)}{\cos }^{3}(\theta )+3{\chi }_{18}^{(3)}\cos (\theta ){\sin }^{2}{(\theta ))}^{2}$$

4 $${I}_{y}^{(3\omega )}\propto ({\chi }_{22}^{(3)}{\sin }^{3}(\theta )+3{\chi }_{29}^{(3)}\sin (\theta ){\cos }^{2}{(\theta ))}^{2}$$

The detected total THG intensity should be expressed as:

5 $$I={I}_{x}+{I}_{y}$$

**Fig. 3:**
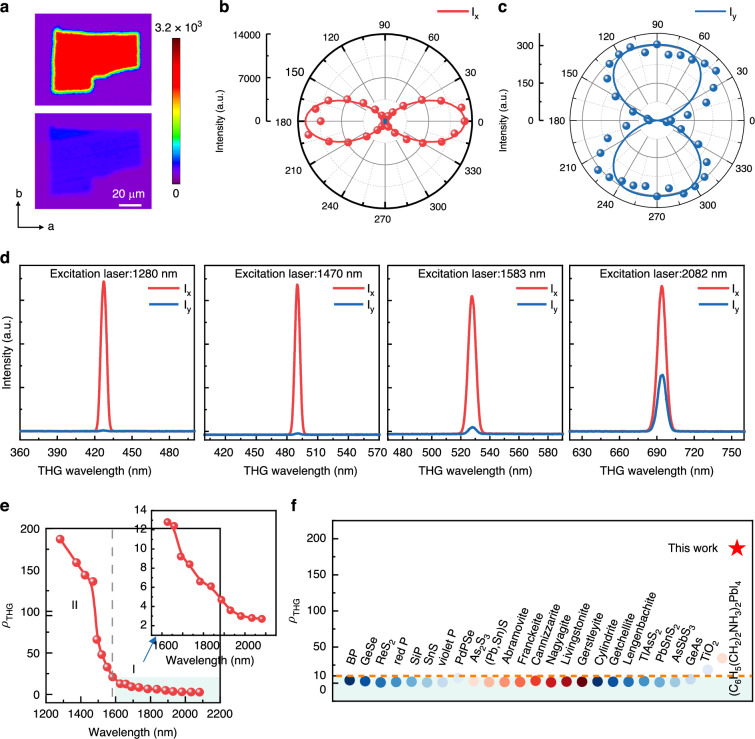
Anisotropic THG process in VOCl flakes. **a** THG mapping for VOCl flakes collected under 1558 nm with parallel (top) and perpendicular (bottom) geometry. **b**, **c** Angle-dependent polarized-THG spectra at 1558 nm. Red (blue) spots represent the *x* (*y*)-component of the THG process, respectively. 0° indicates the direction along the *x*-axis. The solid lines correspond to theoretical fitting curves, indicating the giant THG anisotropy. **d** Polarized *x*(*y*)-component THG spectra (*I*_x_ and *I*_y_) collected under different excitation wavelength (from left to right: 1280, 1470, 1583, 2082 nm, respectively). **e** THG anisotropic ratio as a function of excitation wavelength for a few-layer VOCl flake. The maximum THG anisotropic ratio is ~187 under 1280 nm, the highest reported value. **f** Comparison of THG anisotropic ratios for VOCl and other materials^2,15,53–65^. The shaded region indicates that the THG anisotropic ratio is below 10

As shown in Fig. S14, the total THG intensity vs. polarization angle was explored, revealing giant nonlinear optical anisotropy with two-fold symmetry. Subsequently, the polarized THG images were collected without a polarizer in the detection path. As plotted in Fig. 3a, under *b*-axis-aligned pump polarization, the THG signal reaches its maximum. In contrast, under *b*-axis-perpendicular polarization, i.e., parallel to the *a*-axis, the THG signal is at its lowest intensity. This behavior is consistent with the polarized PL and reflection polarization measurements. Figure 3b, c plot the *x*- and *y*-polarized THG responses on the incident polarization angle for the VOCl nanoflake under 1558 nm excitation. Equations (3) and (4) were used to model the measured results theoretically, producing good agreement with the data. Thus, the relative amplitudes of χ_11_, χ_18_, χ_22_, and χ_29_ were extracted with the ratio of χ_11_ : χ_18_ : χ_22_ : χ_29_ = 1:0.05:0.02:0.026 based on 2D VOCl with a thickness of ~35 nm. Furthermore, the THG ellipsometry consistently shows a zero value for the pump polarization angles spanning 0°-90°, indicating a linearly polarized THG response (Fig. S15a). Additionally, the orientation of the THG polarization aligns well with the incident polarization parallel to *x*- and *y*-axes, respectively (Fig. S15b).

The THG anisotropic ratio *ρ*_THG_ is defined by the equation of ${\rho }_{{THG}}=\,\frac{{I}_{x}}{{I}_{y}}$, which is proportional to the squared ratio between ${\chi }_{11}$ and ${\chi }_{22}$ (${\rho }_{{THG}}\propto {\left(\frac{{\chi }_{11}}{{\chi }_{22}}\right)}^{2}$). In this equation, *I*_x_ and *I*_y_ represent the THG intensities corresponding to the excitation polarized parallel to *x*- and *y*-axes (namely, VOCl’s *b*- and *a*-axes, respectively). As shown in Fig. 3b, c, *I*_x_ is much larger than *I*_y_, suggesting a giant THG anisotropic ratio *ρ*_THG_ ~ 43 for VOCl (~ 35 nm) at a 1558 nm excitation wavelength. According to the anisotropic band dispersion and optical selection rules (see Fig. 2g), the excitation wavelength is an effective strategy to modulate the THG anisotropic ratio *ρ*_THG_. Figure 3d demonstrates the THG intensities of the *x*- and *y*-components under selected excitation wavelengths (1280, 1470, 1583, 2082 nm), implying effective modulation of *ρ*_THG_ by varying the excitation wavelength. Figure 3e illustrates the dependence of *ρ*_THG_ on the excitation wavelength. It is noteworthy that *ρ*_THG_ reaches a maximum value of ~187 under 1280 nm excitation, which is, to date, the largest reported value compared to other materials (see Fig. 3f) observed by far. This underscores the substantial leverage that the excitation wavelength exerts on *ρ*_THG_, enabling up to a 72-fold modulation, providing strong support for the application of 2D VOCl across a broad wavelength range. Moreover, the *ρ*_THG_ behavior can be divided into two distinct regimes. In phase **I** (2082 nm down to ~1550 nm), *ρ*_THG_ gradually increases, whereas in phase **II** (~1550 to 1280 nm), *ρ*_THG_ rises sharply as the wavelength decreases. The striking difference in wavelength-dependent anisotropy between the two phases will be elucidated in the following theoretical analyses. Additionally, the thickness dependence of *ρ*_THG_ was also explored under a 1558 nm excitation wavelength (see Fig. S16). As the thickness increases, *ρ*_THG_ declines, which may be attributed to two factors. First, quantum confinement in ultrathin VOCl gives rise to more localized interband transitions concentrated along one in-plane direction, producing an anisotropic band structure and high THG anisotropy. As the thickness grows (i.e., through interlayer stacking), this confinement diminishes, broadening the electronic states that shape the optical response and thereby reducing anisotropy. Although VOCl exhibits interlayer electronic coupling (its bandgap and χ^(3)^ remain nearly unchanged with the thickness), the stacking nonetheless introduces slight mixing of layer-specific local fields and transitions, gradually enhancing isotropy in thicker samples. In essence, thicker flakes adopt a more three-dimensional arrangement, creating an “averaged” in-plane optical response that raises isotropy and thus lowers *ρ*_THG_.

**Fig. 4:**
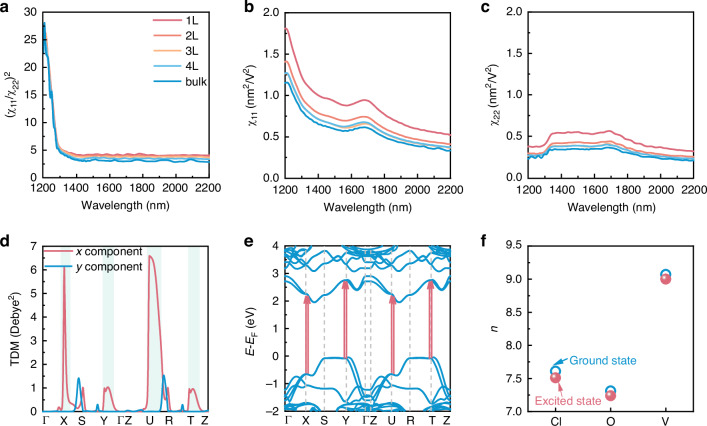
Interpretation of the large in-plane anisotropy of THG in layered VOCl. **a** ${\left(\frac{{\chi }_{11}}{{\chi }_{22}}\right)}^{2}$ for 1 ~ 4 layers and bulk VOCl. **b**, **c** The THG magnitude susceptibilities of ${\chi }_{11}$ and ${\chi }_{22}$ components for 1 ~ 4 la*y*ers and bulk VOCl. **d** The *x*- and *y*-component transition dipole moments. The light blue shaded regions indicate the optical transitions that contribute to the THG around 1200 nm. **e** The optical transitions contributing to the THG anisotropic ratio peak located around the X, Y, U and T high symmetry *k*-point, as indicated by red arrows, and corresponding to the transition vector labeled in (**d**). **f** The ground- and excited-state charge populations on V, O, and Cl atoms

To unveil the underlying mechanism of the high THG anisotropy observed in the 2D VOCl, we calculated its THG response using a tight-binding Hamiltonian derived from DFT wavefunctions. Based on the relationship of ${\rho }_{{THG}}\propto {\left(\frac{{\chi }_{11}}{{\chi }_{22}}\right)}^{2}$, Fig. 4a shows ${\left(\frac{{\chi }_{11}}{{\chi }_{22}}\right)}^{2}$ for 1 L to 4 L and bulk VOCl, where the tendency of the ratio with the excitation wavelengths shows a good agreement with our experimental observations (see Fig. 3e). As excitation wavelength shortens (but remains in phase **I**), both χ_11_ and χ_22_ all increase (see Fig. 4b and c), yet their ratio ${\left(\frac{{\chi }_{11}}{{\chi }_{22}}\right)}^{2}$ remains modest (only a few to tens), accounting for the weak anisotropy in phase **I** of Fig. 3e. Here, the third-harmonic photon energy in phase **I** (~ 1.69-2.30 eV) lies below the interband transition, thus unable to trigger charge transfer from O_*p* and Cl_*p* to V_*d* orbitals (Figs. 1a and d–f), leading to a small *ρ*_THG_. By contrast, once the excitation wavelength decreases further (phase **II**, ~ 2.3-3.0 eV), χ_11_ increases rapidly while χ_22_ remains unchanged or even diminishes, causing ${\left(\frac{{\chi }_{11}}{{\chi }_{22}}\right)}^{2}$ to rise sharply and peak around 1200 nm (see Fig. 4a-c). To reveal the origin of the high THG anisotropy, the transition dipole moment (TDM) was calculated along a high symmetric *k*-point, as shown in Fig. 4d. The optical transitions demonstrate a large anisotropy between the *x* and *y* components around the X, Y, U, and T points, marked with light blue shaded regions. The large and expanded TDM in the *x* direction corresponds to the band transitions indicated by red arrows (see Fig. 4e), explaining the swift increase in *ρ*_THG_ during phase **II** and its peak near 1200 nm. From the orbital-projected band structure, the transitions primarily occur from V_*d*_xy_, O_*p*_x_, Cl_*p*_y_ orbitals to V_*d*_yz_ orbitals (see Fig. 1d–f) ($|{d}_{xy},{p}_{y}\rangle +|{d}_{xy},{p}_{x}\rangle \to |{d}_{yz}\rangle$), accompanied by a ~ 3.0 eV charge-transfer gap matching the THG response at ~1200 nm. Based on the composition of the orbital-resolved band structure, we define layered VOCl as a typical CTMI (Fig. 1a), which is regarded as a potential candidate for generating strong nonlinear optical responses and significant nonlinear optical anisotropy.^24^ To clarify the role of charge transfer in the THG process and its anisotropy, the charge populations of the ground state and excited state on each atom were extracted, as shown in Fig. 4f. An evident decrease in charge population is observed in the excited state relative to the ground state. Moreover, the decrease in charge population of the Cl and O atoms is larger than that of the V atom, signifying a substantial *p* → *d* transfer at resonance energies around 3.0 eV, consistent with the slight *p*-type conductivity in VOCl revealed by electrical transport measurements (Fig. S17). Thus, combining the analyses of orbital-resolved band structures and optical transitions (see Figs. 1d–f and 4d–f), it can be concluded that when the photon energy is tuned to the scale of the charge-transfer gap, a significant charge transfer occurs in the excited state between *p*-orbitals and *d*-orbitals, driving a rapid rise and exceptionally high THG anisotropy near 1200 nm. Excitation-induced charge transfer preserves the ground-state antiferromagnetic ordering, therefore, the giant THG ratio and THG response present a strong correlation with such separate phases.

## Discussion

In brief, we report a 2D CTMI VOCl that exhibits substantial linear and nonlinear optical anisotropy. First-principle calculations were applied to demonstrate the correlated Mott insulator nature for 2D VOCl. The magnetic properties were measured to emphasize its antiferromagnetic features. Comprehensive characterizations were utilized to demonstrate the linear and nonlinear optical properties, illustrating giant anisotropy ratios in PL and THG, with values of 6.7 and 187, respectively. It is worth noting that this is the largest THG anisotropy ratio reported to date. Furthermore, the THG anisotropic ratio can be significantly enhanced from 2.6 to 187 as the excitation wavelength shifts from 2028 to 1280 nm, resulting in 72 times improvement. The giant, tunable THG anisotropy of VOCl makes them promising building blocks for integrated polarization beam splitters, polarized long-wave upconversion photodetector, and polarized ultrafast lasers, opening new avenues for next-generation polarization-engineered nanophotonic devices. Furthermore, theoretical calculations indicate that the giant THG anisotropy can be explained by the synergistic effect of the CTMI behavior and *C*_3_ symmetry breaking. Besides, VOCl exhibits extremely weak interlayer coupling, as demonstrated by its thickness-independent band gap and χ^(3)^. Taking a broader view, our result provides a pathway for 2D correlated Mott insulators in nanophotonic and optoelectronic applications.

## Materials and methods

### Crystal preparation

Bulk crystals VOCl were synthesized via a CVT approach. A quartz ampoule was loaded with 0.5 g of V, V_2_O_5_, and VCl_3_ powder (molar ratio of 4:3:5), evacuated to < 10^-2 ^Pa, flame-sealed, and subsequently positioned in the center of a horizontal double-zone furnace. Then, the two ends of the furnace were heated to 850 °C and 950 °C over 30 hours, respectively. After two weeks, bulk crystals with a brown color were obtained in the cold zone. The obtained single crystals were mechanically exfoliated onto a SiO_2_/Si substrate (285 nm SiO_2_ film), leaving VOCl nanoflakes with different thicknesses and sizes on the substrate.

### Materials characterization

Powder XRD measurement was performed on a Bruker D8 Advance powder diffractometer (*λ* = 1.5406 Å, Cu−Kα radiation). Elemental analysis of the bulk crystals was conducted via energy-dispersive X-ray spectroscopy (EDX, Oxford INCA) integrated into a field emission scanning electron microscope (FESEM, JEOL JSM-7600F). XPS measurements were carried out using a Kratos AXIS Supra equipped with a dual-anode Al-Kα (1486.6 eV) monochromator, and energies were referenced to C1s = 284.8 eV. AFM imaging was carried out on an Asylum Research Cypher S and a Veeco Dimension 3100. The atomic structure of exfoliated VOCl flakes was recorded using a modified JEOL 2100 F scanning transmission electron microscope (STEM). PL and Raman spectra were obtained with aid of a WITec CRM200 confocal system (*λ*_ex_ = 532 nm). Absorption was determined by comparing the transmission through VOCl flakes with that of a fused silica substrate. Reflection spectrum was obtained with a Fourier transform spectrometer featuring a cooled mercury- cadmium-telluride (MCT) detector.

## THG process measurements

THG process was evaluated by a custom-designed optical setup capable of operating in both reflective and transmissive configurations.^2,29^ The nonlinear optical responses were probed with a femtosecond laser system tunable over 650-2600 nm, delivering 250 fs pulses at 100 kHz repetition rate. The excitation laser was directed through a neutral density (ND) filter, a polarizer, a half-wave plate, and a NA 0.45, 50× objective lens, resulting in a ~ 3 μm focused spot size. The transmitted laser was obtained by a NA = 0.45, 20× objective lens, while a short-pass filter was employed to suppress the excitation light. The nonlinear optical process was subsequently collected by a spectrometer equipped with a cooled detector. All THG process measurements are collected at room temperature.

## First-principles calculations

The simulations of the third harmonic generation (THG) in layered VOCl were performed using density functional theory (DFT) coupled to Wannier-based interpolation. In DFT calculations, a Hubbard correction of U = 4.5 eV was adopted to obtain the nature of the semiconductor for VOCl within the framework of PBE-GGA functional^46^ implemented in VASP package^47,48^. Structural relaxations were deemed converged when the absolute force value on any atom was < 0.01 eV/ Å. optB86b-vdW functional^49,50^ scheme was utilized to assess van der Waals interlayer interactions. Γ-centered Brillouin-zone samplings of 15 × 15 × 3 (bulk) and 15 × 15 × 1 (layered VOCl) were adopted. The cutoff energy was set to 500 eV and spin-orbit coupling was included for all DFT calculations. Using maximally localized Wannier functions, the tight-binding Hamiltonian was constructed using Wannier90 package^51^ to reproduce the band structure of DFT.

Based on the constructed tight-binding Hamiltonian, the calculations of third-order optical response were implemented in ERETCAD package. Nonlinear susceptibility (${\chi }^{(3)}$) is composed of interband contribution (${\chi }_{\chi }^{(3)}$) and intraband contribution (${\chi }_{\sigma }^{(3)}$)^52^:

6 $${\chi }^{(3)}={\chi }_{\chi }^{(3)}+{\chi }_{\sigma }^{(3)}$$

Alternatively, the interband contribution is described as:

7 $$\begin{array}{rcl}\frac{{\chi }_{\chi }^{(3)}}{C} & = & \mathop{\sum }\limits_{l,m,n,p,{\bf{k}}}\frac{{r}_{mn}^{d}}{{\omega }_{nm}-{\omega }_{3}}\left[\frac{{r}_{nl}^{c}}{{\omega }_{lm}-{\omega }_{2}}\left(\frac{{r}_{lp}^{b}{r}_{pm}^{a}{f}_{mp}}{{\omega }_{pm}-{\omega }_{1}}-\frac{{r}_{lp}^{a}{r}_{pm}^{b}{f}_{pl}}{{\omega }_{lp}-{\omega }_{1}}\right)-\frac{{r}_{pm}^{c}}{{\omega }_{np}-{\omega }_{2}}\left(\frac{{r}_{nl}^{b}{r}_{lp}^{a}{f}_{pl}}{{\omega }_{lp}-{\omega }_{1}}-\frac{{r}_{nl}^{a}{r}_{lp}^{b}{f}_{\mathrm{ln}}}{{\omega }_{nl}-{\omega }_{1}}\right)\right]\\ & & +i{\mathop{\sum }\limits_{l,m,n,{\bf{k}}}\frac{{r}_{mn}^{d}}{{\omega }_{nm}-{\omega }_{3}}\left[\frac{1}{{\omega }_{nm}-{\omega }_{2}}\left(\frac{{r}_{nl}^{b}{r}_{lm}^{a}{f}_{ml}}{{\omega }_{lm}-{\omega }_{1}}-\frac{{r}_{nl}^{a}{r}_{lm}^{b}{f}_{ln}}{{\omega }_{nl}-{\omega }_{1}}\right)\right]}_{;{k}^{c}}\\ & & +i\mathop{\sum }\limits_{l,m,n,{\bf{k}}}\frac{{r}_{mn}^{d}}{{\omega }_{nm}-{\omega }_{3}}\left[\frac{{r}_{nl}^{c}}{{\omega }_{lm}-{\omega }_{2}}{\left(\frac{{r}_{lm}^{a}{f}_{ml}}{{\omega }_{lm}-{\omega }_{1}}\right)}_{;{k}^{b}}-\frac{{r}_{lm}^{c}}{{\omega }_{nl}-{\omega }_{1}}{\left(\frac{{r}_{nl}^{a}{f}_{ln}}{{\omega }_{nl}-{\omega }_{1}}\right)}_{;{k}^{b}}\right]\\ & & -\mathop{\sum }\limits_{m,n,{\bf{k}}}\frac{{r}_{mn}^{f}}{{\omega }_{nm}-{\omega }_{3}}{\left[\frac{1}{{\omega }_{nm}-{\omega }_{2}}{\left(\frac{{r}_{nm}^{a}{f}_{nm}}{{\omega }_{nm}-{\omega }_{1}}\right)}_{;{k}^{b}}\right]}_{;{k}^{c}}\end{array}$$

The intraband contribution is described as:

8 $$\begin{array}{l}\frac{{\chi }_{\sigma }^{(3)}}{C}=\frac{1}{i{\omega }_{3}^{2}}\mathop{\sum }\limits_{l,m,n,{\bf{k}}}{\varDelta }_{nl}^{d}\frac{{r}_{nl}^{c}}{{\omega }_{ln}-{\omega }_{2}}\left(\frac{{r}_{lm}^{b}{r}_{mn}^{a}{f}_{nm}}{{\omega }_{mn}-{\omega }_{1}}-\frac{{r}_{lm}^{a}{r}_{mn}^{b}{f}_{ml}}{{\omega }_{lm}-{\omega }_{1}}\right)-\frac{1}{{\omega }_{3}^{2}{\omega }_{2}}{\mathop{\sum }\limits_{m,n,{\bf{k}}}{\varDelta }_{nm}^{d}\left(\frac{{r}_{nm}^{b}{r}_{mn}^{a}{f}_{nm}}{{\omega }_{mn}-{\omega }_{1}}\right)}_{;{k}^{c}}\\ \begin{array}{rcl} & & +\end{array}\frac{1}{{\omega }_{3}^{2}}{\mathop{\sum }\limits_{m,n,{\bf{k}}}{\varDelta }_{nm}^{d}\frac{{r}_{nm}^{c}}{{\omega }_{mn}-{\omega }_{2}}\left(\frac{{r}_{mn}^{a}{f}_{nm}}{{\omega }_{mn}-{\omega }_{1}}\right)}_{;{k}^{b}}-\frac{i}{{\omega }_{3}}\mathop{\sum }\limits_{m,n,l,{\bf{k}}}\frac{{({r}_{nl}^{c})}_{;{k}^{d}}}{{\omega }_{mn}-{\omega }_{2}}\left(\frac{{r}_{lm}^{b}{r}_{mn}^{a}{f}_{nm}}{{\omega }_{mn}-{\omega }_{1}}-\frac{{r}_{lm}^{a}{r}_{mn}^{b}{f}_{ml}}{{\omega }_{lm}-{\omega }_{1}}\right)\\ \begin{array}{rcl} & & +\frac{1}{{\omega }_{3}}\mathop{\sum }\limits_{m,n,{\bf{k}}}\frac{{({r}_{nm}^{c})}_{;{k}^{d}}}{{\omega }_{mn}-{\omega }_{2}}{\left(\frac{{r}_{mn}^{a}{f}_{nm}}{{\omega }_{mn}-{\omega }_{1}}\right)}_{;{k}^{b}}-\end{array}\frac{1}{i{\omega }_{3}{\omega }_{2}}\mathop{\sum }\limits_{m,n,{\bf{k}}}\frac{{r}_{nm}^{b}{r}_{mn}^{a}{f}_{nm}}{{\omega }_{mn}-{\omega }_{1}}\left(\frac{\partial {\varepsilon }_{nm}^{c}}{\partial {k}^{d}}-\frac{\partial {\varepsilon }_{nm}^{d}}{\partial {k}^{c}}\right)\end{array}$$

where$C={e}^{3}K/{\hslash }^{2}m$ with *K* accounting for the usual factors, *r* is the position operator, ${\omega }_{mn}$ is the frequency offset between bands *m* and *n*, and *f* denotes the Fermi-Dirac distribution. Furthermore, the optical transition dipole moment is evaluated from the constructed wavefunctions,

9 $${{\rm{TDM}}}_{a\to b}=\frac{i\hslash }{({E}_{b}-{E}_{a})m}\langle {\psi }_{a}|{\bf{p}}|{\psi }_{b}\rangle =e\int {\psi }_{b}^{\ast }({\bf{r}}){\bf{r}}{\psi }_{a}({\bf{r}}){{\rm{d}}}^{3}{\bf{r}}$$

where *ψ*_*a*_ and *ψ*_*b*_ are the wavefunctions of eigenstates *E*_*a*_ and *E*_*b*_, respectively.

## Supplementary information

Supplementary Information for Colossal infrared nonlinear optical anisotropy in a 2D charge-transfer Mott insulator

## References

[1] Yue, L. et al. Giant nonlinear optical wave mixing in a van der Waals correlated insulator. *Sci. Adv.* **10**, eadn6216 (2024). 39093978 10.1126/sciadv.adn6216 PMC11296339

[2] Zhu, S. et al. Strong nonlinear optical processes with extraordinary polarization anisotropy in inversion-symmetry broken two-dimensional PdPSe. *Light Sci. Appl.* **13**, 119 (2024). 38802363 10.1038/s41377-024-01474-6 PMC11130276

[3] Guo, Q. B. et al. Colossal in-plane optical anisotropy in a two-dimensional van der Waals crystal. *Nat. Photonics* **18**, 1170–1175 (2024).

[4] Zhou, Y. et al. A solution-processable natural crystal with giant optical anisotropy for efficient manipulation of light polarization. *Nat. Photonics* **18**, 922–927 (2024).

[5] Autere, A. et al. Nonlinear optics with 2D layered materials. *Adv. Mater.* **30**, 1705963 (2018). 10.1002/adma.201705963 29575171

[6] Ma, R., Sutherland, D. S. & Shi, Y. M. Harmonic generation in transition metal dichalcogenides and their heterostructures. *Mater. Today* **50**, 570–586 (2021).

[7] Xie, Z. X. et al. Nonlinear optical properties of 2D materials and their applications. *Small* **20**, 2311621 (2024). 10.1002/smll.202311621 38618662

[8] Nicholls, L. H. et al. Ultrafast synthesis and switching of light polarization in nonlinear anisotropic metamaterials. *Nat. Photonics* **11**, 628–633 (2017).

[9] Wu, L. et al. Giant anisotropic nonlinear optical response in transition metal monopnictide Weyl semimetals. *Nat. Phys.* **13**, 350–355 (2017).

[10] Niu, S. Y. et al. Giant optical anisotropy in a quasi-one-dimensional crystal. *Nat. Photonics* **12**, 392–396 (2018).

[11] Zhang, H. Q. et al. Cavity-enhanced linear dichroism in a van der Waals antiferromagnet. *Nat. Photonics* **16**, 311–317 (2022).

[12] Wu, C. et al. Giant optical anisotropy in the UV-transparent 2D nonlinear optical material Sc(IO_3_)_2_(NO_3_). *Angew. Chem. Int. Ed.* **60**, 3464–3468 (2021). 10.1002/anie.202012456 33146456

[13] Li, X. et al. Review of anisotropic 2D materials: controlled growth, optical anisotropy modulation, and photonic applications. *Laser Photonics Rev.* **15**, 2100322 (2021).

[14] Ermolaev, G. A. et al. Giant optical anisotropy in transition metal dichalcogenides for next-generation photonics. *Nat. Commun.* **12**, 854 (2021). 33558559 10.1038/s41467-021-21139-x PMC7870936

[15] Du, L. J. et al. Giant anisotropic photonics in the 1D van der Waals semiconductor fibrous red phosphorus. *Nat. Commun.* **12**, 4822 (2021). 34376660 10.1038/s41467-021-25104-6 PMC8355160

[16] Ghosh, H. Excited state nonlinear optics of quasi-one-dimensional Mott-Hubbard insulators. *Phys. Rev. B* **75**, 235127 (2007).

[17] Kishida, H. et al. Gigantic optical nonlinearity in one-dimensional Mott–Hubbard insulators. *Nature* **405**, 929–932 (2000). 10879529 10.1038/35016036

[18] Murakami, Y. et al. Anomalous temperature dependence of high-harmonic generation in Mott insulators. *Phys. Rev. Lett.* **129**, 157401 (2022). 36269969 10.1103/PhysRevLett.129.157401

[19] Uchida, K. et al. High-order harmonic generation and its unconventional scaling law in the Mott-insulating Ca_2_RuO_4_. *Phys. Rev. Lett.* **128**, 127401 (2022). 35394320 10.1103/PhysRevLett.128.127401

[20] Hubbard, J. Electron correlations in narrow energy bands. *Proc. R. Soc. A* **276**, 238–257 (1963).

[21] Imada, M., Fujimori, A. & Tokura, Y. Metal-insulator transitions. *Rev. Mod. Phys.* **70**, 1039–1263 (1998).

[22] Takahashi, M., Tohyama, T. & Maekawa, S. Nonlinear optical response in two-dimensional Mott insulators. *Phys. Rev. B* **66**, 125102 (2002).

[23] Ono, M. et al. Linear and nonlinear optical properties of one-dimensional Mott insulators consisting of Ni-halogen chain and CuO-chain compounds. *Phys. Rev. B* **70**, 085101 (2004).

[24] Zhang, G. P. Origin of giant optical nonlinearity in charge-transfer-Mott insulators: a new paradigm for nonlinear optics. *Phys. Rev. Lett.* **86**, 2086–2089 (2001). 11289861 10.1103/PhysRevLett.86.2086

[25] Boyd, R. W. *Nonlinear Optics*. 10.1016/C2015-0-05510-1 (Academic, 2020).

[26] Li, G. C. et al. Light-induced symmetry breaking for enhancing second-harmonic generation from an ultrathin plasmonic nanocavity. *Nat. Commun.* **12**, 4326 (2021). 34267205 10.1038/s41467-021-24408-x PMC8282679

[27] Glawion, S. et al. Electronic structure of the two-dimensional Heisenberg antiferromagnet VOCl: a multiorbital Mott insulator. *Phys. Rev. B* **80**, 155119 (2009).

[28] Villalpando, G. et al. Chemical exfoliation toward magnetic 2D VOCl monolayers. *ACS Nano* **16**, 13814–13820 (2022). 35977071 10.1021/acsnano.2c01858

[29] Zhu, S. et al. Ultrastrong optical harmonic generations in layered platinum disulfide in the mid-infrared. *ACS Nano* **17**, 2148–2158 (2023). 36706067 10.1021/acsnano.2c08147

[30] Autere, A. et al. Rapid and large-area characterization of exfoliated black phosphorus using third-harmonic generation microscopy. *J. Phys. Chem. Lett.* **8**, 1343–1350 (2017). 28266862 10.1021/acs.jpclett.7b00140

[31] Woodward, R. I. et al. Characterization of the second- and third-order nonlinear optical susceptibilities of monolayer MoS_2_ using multiphoton microscopy. *2D Mater.* **4**, 011006 (2017).

[32] Autere, A. et al. Optical harmonic generation in monolayer group-VI transition metal dichalcogenides. *Phys. Rev. B* **98**, 115426 (2018).

[33] Zhu, W. et al. Ternary VOCl single-crystal as efficient gate dielectric for 2D field-effect transistors. *2D Mater.* **8**, 025010 (2021).

[34] Oyedele, A. D. et al. PdSe_2_: pentagonal two-dimensional layers with high air stability for electronics. *J. Am. Chem. Soc.* **139**, 14090–14097 (2017). 28873294 10.1021/jacs.7b04865

[35] Yu, X. C. et al. Atomically thin noble metal dichalcogenide: a broadband mid-infrared semiconductor. *Nat. Commun.* **9**, 1545 (2018). 29670119 10.1038/s41467-018-03935-0 PMC5906448

[36] Das, S. et al. Tunable transport gap in phosphorene. *Nano Lett.* **14**, 5733–5739 (2014). 25111042 10.1021/nl5025535

[37] Zaanen, J., Sawatzky, G. A. & Allen, J. W. Band gaps and electronic structure of transition-metal compounds. *Phys. Rev. Lett.* **55**, 418–421 (1985). 10032345 10.1103/PhysRevLett.55.418

[38] Gao, S. Y. et al. Discovery of a single-band Mott insulator in a van der Waals flat-band compound. *Phys. Rev. X* **13**, 041049 (2023).

[39] Tang, E. et al. High-temperature fractional quantum Hall states. *Phys. Rev. Lett.* **106**, 236802 (2011). 21770532 10.1103/PhysRevLett.106.236802

[40] Du, L. J. et al. Engineering symmetry breaking in 2D layered materials. *Nat. Rev. Phys.* **3**, 193–206 (2021).

[41] Cai, H. et al. Synthesis of highly anisotropic semiconducting GaTe nanomaterials and emerging properties enabled by epitaxy. *Adv. Mater.* **29**, 1605551 (2017). 10.1002/adma.201605551 27990702

[42] Aslan, B. et al. Linearly polarized excitons in single- and few-layer ReS_2_ crystals. *ACS Photonics* **3**, 96–101 (2016).

[43] Khatibi, A. et al. Anisotropic infrared light emission from quasi-1D layered TiS_3_. *2D Mater.* **7**, 015022 (2020).

[44] Zhou, Z. Q. et al. Perpendicular optical reversal of the linear dichroism and polarized photodetection in 2D GeAs. *ACS Nano* **12**, 12416–12423 (2018). 30408410 10.1021/acsnano.8b06629

[45] Yang, X. L. & Xie, S. W. Expression of third-order effective nonlinear susceptibility for third-harmonic generation in crystals. *Appl. Opt.* **34**, 6130–6135 (1995). 21060454 10.1364/AO.34.006130

[46] Perdew, J. P., Burke, K. & Ernzerhof, M. Generalized gradient approximation made simple. *Phys. Rev. Lett.* **77**, 3865–3868 (1996). 10062328 10.1103/PhysRevLett.77.3865

[47] Kresse, G. & Furthmüller, J. Efficient iterative schemes for ab initio total-energy calculations using a plane-wave basis set. *Phys. Rev. B* **54**, 11169–11186 (1996). 10.1103/physrevb.54.11169 9984901

[48] Kresse, G. & Furthmüller, J. Efficiency of ab-initio total energy calculations for metals and semiconductors using a plane-wave basis set. *Computational Mater. Sci.* **6**, 15–50 (1996). 10.1103/physrevb.54.11169 9984901

[49] Klimeš, J., Bowler, D. R. & Michaelides, A. Chemical accuracy for the van der Waals density functional. *J. Phys.: Condens. Matter* **22**, 022201 (2010). 21386245 10.1088/0953-8984/22/2/022201

[50] Klimeš, J., Bowler, D. R. & Michaelides, A. Van der Waals density functionals applied to solids. *Phys. Rev. B* **83**, 195131 (2011).

[51] Marzari, N. et al. Maximally localized Wannier functions: theory and applications. *Rev. Mod. Phys.* **84**, 1419–1475 (2012).

[52] Aversa, C. & Sipe, J. E. Nonlinear optical susceptibilities of semiconductors: results with a length-gauge analysis. *Phys. Rev. B* **52**, 14636–14645 (1995). 10.1103/physrevb.52.14636 9980796

[53] Xia, C. S. et al. Strong anisotropic third-harmonic generation in layered violet phosphorus. *Nanoscale* **16**, 11663–11668 (2024). 38853705 10.1039/d4nr01337a

[54] Maragkakis, G. M. et al. Anisotropic third harmonic generation in 2D tin sulfide. *Adv. Optical Mater.* **12**, 2401321 (2024).

[55] Sar, H., Gao, J. & Yang, X. D. 2D layered SiP as anisotropic nonlinear optical material. *Sci. Rep.* **11**, 6372 (2021). 33737690 10.1038/s41598-021-85938-4 PMC7973522

[56] Dasgupta, A., Gao, J. & Yang, X. D. Anisotropic third-harmonic generation in layered germanium selenide. *Laser Photonics Rev.* **14**, 1900416 (2020).

[57] Cui, Q. N. et al. Strong and anisotropic third-harmonic generation in monolayer and multilayer ReS_2_. *Phys. Rev. B* **95**, 165406 (2017).

[58] Sar, H., Gao, J. & Yang, X. D. In-plane anisotropic third-harmonic generation from germanium arsenide thin flakes. *Sci. Rep.* **10**, 14282 (2020). 32868859 10.1038/s41598-020-71244-y PMC7458918

[59] Youngblood, N. et al. Layer-tunable third-harmonic generation in multilayer black phosphorus. *ACS Photonics* **4**, 8–14 (2017).

[60] Dasgupta, A., Gao, J. & Yang, X. D. Natural van der Waals heterostructure cylindrite with highly anisotropic optical responses. *npj 2D Mater. Appl.* **5**, 74 (2021).

[61] Dasgupta, A., Yang, X. D. & Gao, J. Naturally occurring van der Waals heterostructure lengenbachite with strong in-plane structural and optical anisotropy. *npj 2D Mater. Appl.* **5**, 88 (2021).

[62] Tripathi, R. P. N., Yang, X. D. & Gao, J. Van der Waals layered mineral getchellite with anisotropic linear and nonlinear optical responses. *Laser Photonics Rev.* **15**, 2100182 (2021).

[63] Tripathi, R. P. N., Gao, J. & Yang, X. D. Naturally occurring layered mineral franckeite with anisotropic Raman scattering and third-harmonic generation responses. *Sci. Rep.* **11**, 8510 (2021). 33875773 10.1038/s41598-021-88143-5 PMC8055868

[64] Dasgupta, A. et al. Large in-plane vibrational and optical anisotropy in natural 2D heterostructure abramovite. *Sci. Rep.* **12**, 16803 (2022). 36207449 10.1038/s41598-022-21042-5 PMC9547020

[65] Chen, Z. H. et al. In-plane anisotropic nonlinear optical properties of two-dimensional organic–inorganic hybrid perovskite. *J. Phys. Chem. Lett.* **12**, 7010–7018 (2021). 34286998 10.1021/acs.jpclett.1c01890

