# Supplementary material for: Colossal infrared nonlinear optical anisotropy in a 2D charge-transfer Mott insulator
Source: Light Sci Appl. 2026 Jan 8;15:59. doi: 10.1038/s41377-025-02130-3 (PMC12780175; doi:10.1038/s41377-025-02130-3)
Supplement: Supplementary file 1 — Supplementary Information for Colossal infrared nonlinear optical anisotropy in a 2D charge-transfer Mott insulator [file 41377_2025_2130_MOESM1_ESM.docx]

Supplementary Information for

Colossal infrared nonlinear optical anisotropy in a 2D charge-transfer Mott insulator

Ruihuan Duan^1, †^, Song Zhu^2, 3 †^, Xiaodong Xu^4, †^, Yao Wu^1^, Sicheng Zhou^5^, Xuan Mao^2^, Zhen Xu^2^, Wenduo Chen^2^, Xiaodan Lyu^6^, Youqiang Huang^7^, Yi Zhang^7^, Fakun Wang^2^, Lishu Wu^1^, Ya Deng^1^, Manzhang Xu^8^, Yanchao He^1^, Jiayu Shi^1^, Wenting Zhao^1^, Guangtong Liu^5^, Weibo Gao^6^, Zhipei Sun^7^, Xingji Li^4,^ *, Qi Jie Wang^2, 6,^ *, and Zheng Liu^1,^ *

^1^School of Materials Science and Engineering, Nanyang Technological University, 639798, Singapore

^2^School of Electrical and Electronic Engineering, Nanyang Technological University, 639798, Singapore

^3^National Key Laboratory of Microwave Photonics, Nanjing University of Aeronautics and Astronautics, Nanjing, China

^4^School of Materials Science and Engineering, Harbin Institute of Technology, Harbin, 150001, China

^5^Beijing National Laboratory of Condensed Matter Physics, Institute of Physics, Chinese Academy of Sciences, 100190 Beijing, China

^6^School of Physical and Mathematical Sciences, Nanyang Technological University, 637371, Singapore

^7^Department of Electronics and Nanoengineering and QTF Centre of Excellence, Aalto University, Aalto, Finland

^8^Frontiers Science Center for Flexible Electronics (FSCFE) & Institute of Flexible Electronics (IFE), Northwestern Polytechnical University, Xi'an 710072, China.

^†^These authors contributed equally to this work.

*Corresponding authors: [z.liu@ntu.edu.sg](mailto:z.liu@ntu.edu.sg), qjwang@ntu.edu.sg, lxj0218@hit.edu.cn


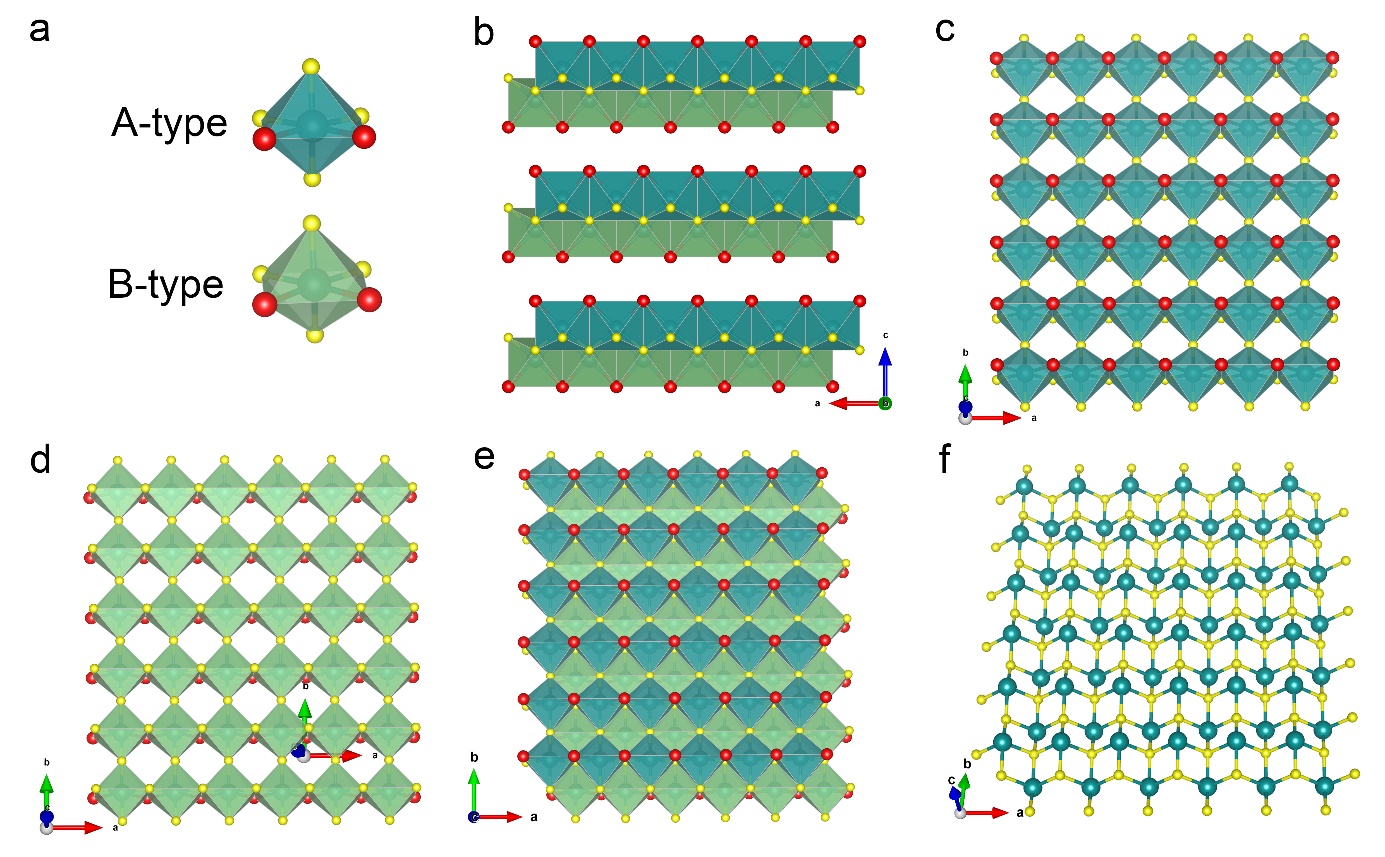


Figure S1. Crystal structure of 2D VOCl. a) A and B-type octahedra. b) Crystal structure of VOCl viewing along the *b*-axis. c) A-type octahedral planes. d) B-type octahedral planes.


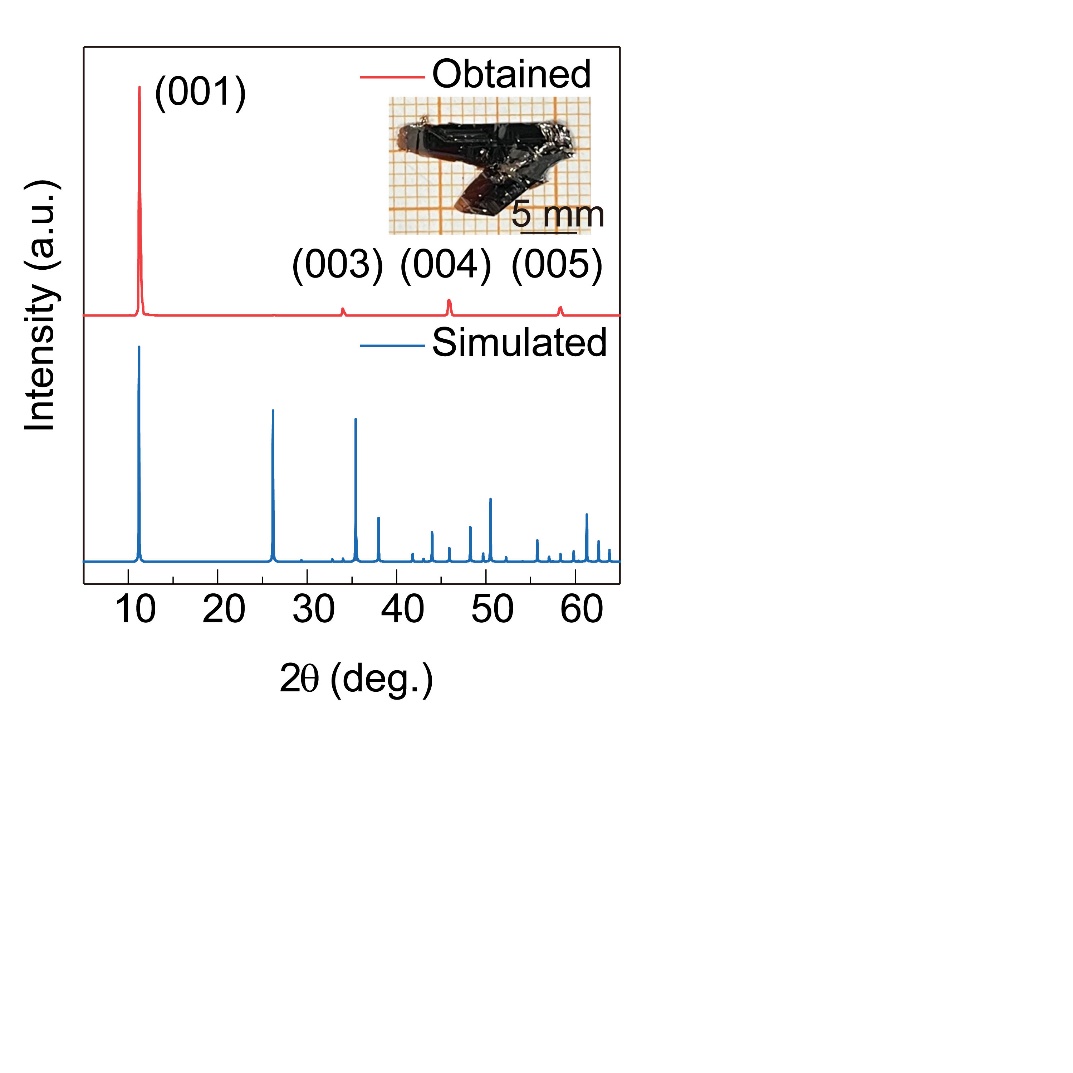


Figure S2. XRD patterns for VOCl. Inset: Optical image for as-grown VOCl crystals with lengths up to 15 mm.


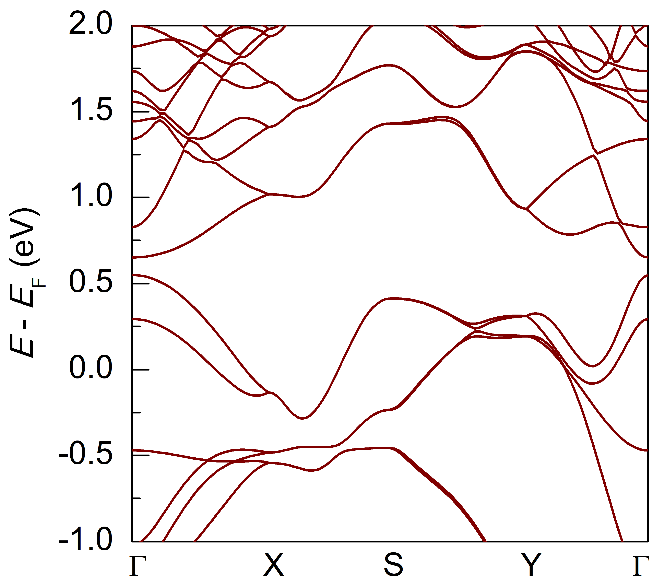


Figure S3. Band structure of monolayer VOCl at GGA-PBE level.


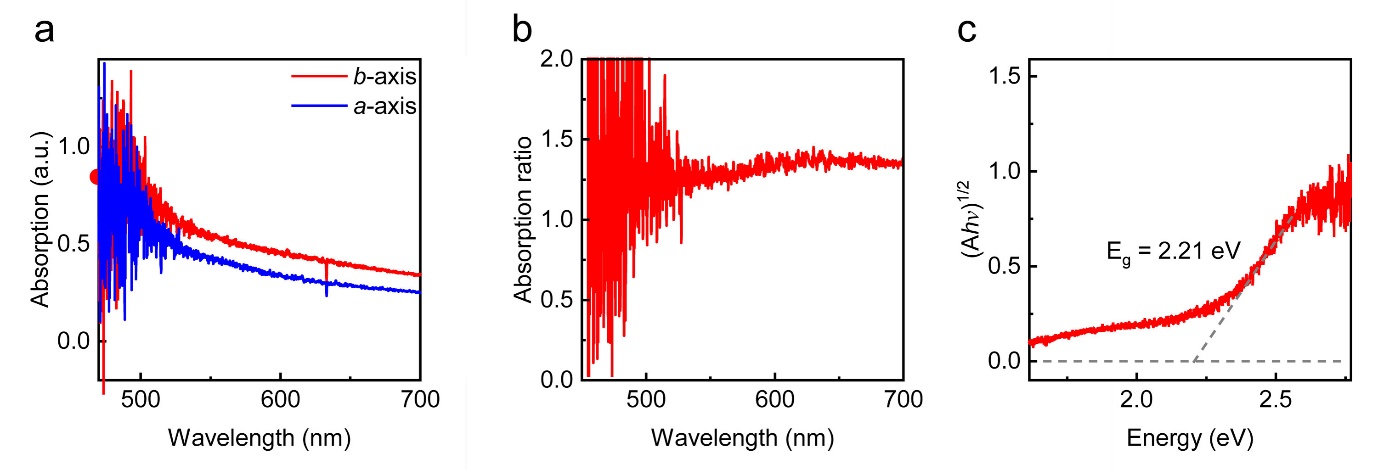


Figure S4. a) Absorption spectra of VOCl for light polarized along the *a*- and *b*-axes, respectively. b) Absorption anisotropic ratio of VOCl, which equals to A*_b_*_-axis_/A*_a_*_-axis_. c) Band gap plot for VOCl flakes.


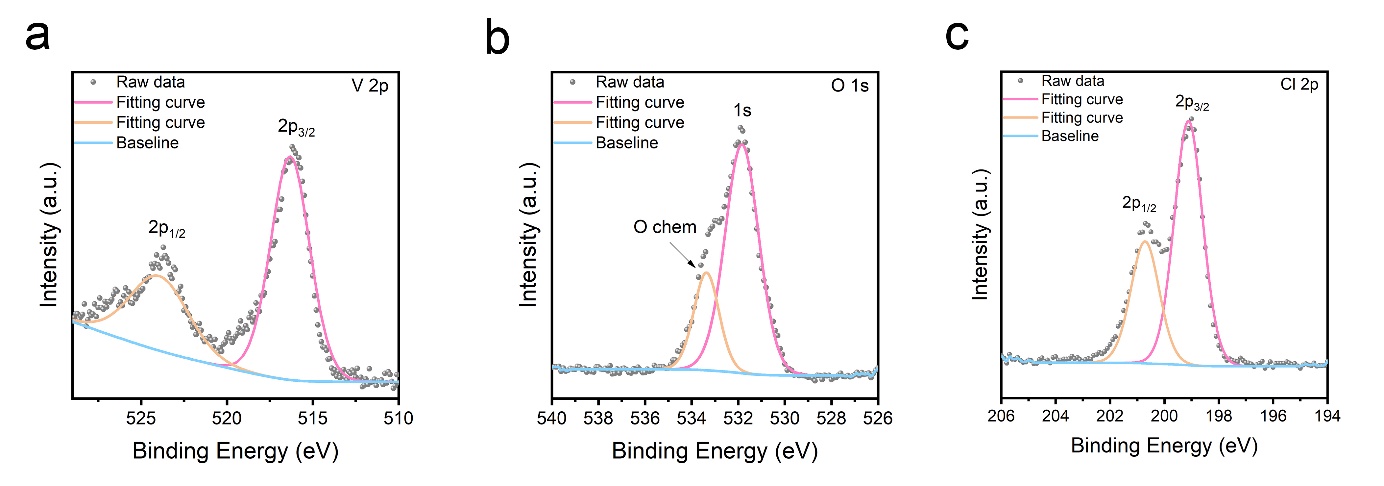


Figure S5. XPS spectra for 2D VOCl.


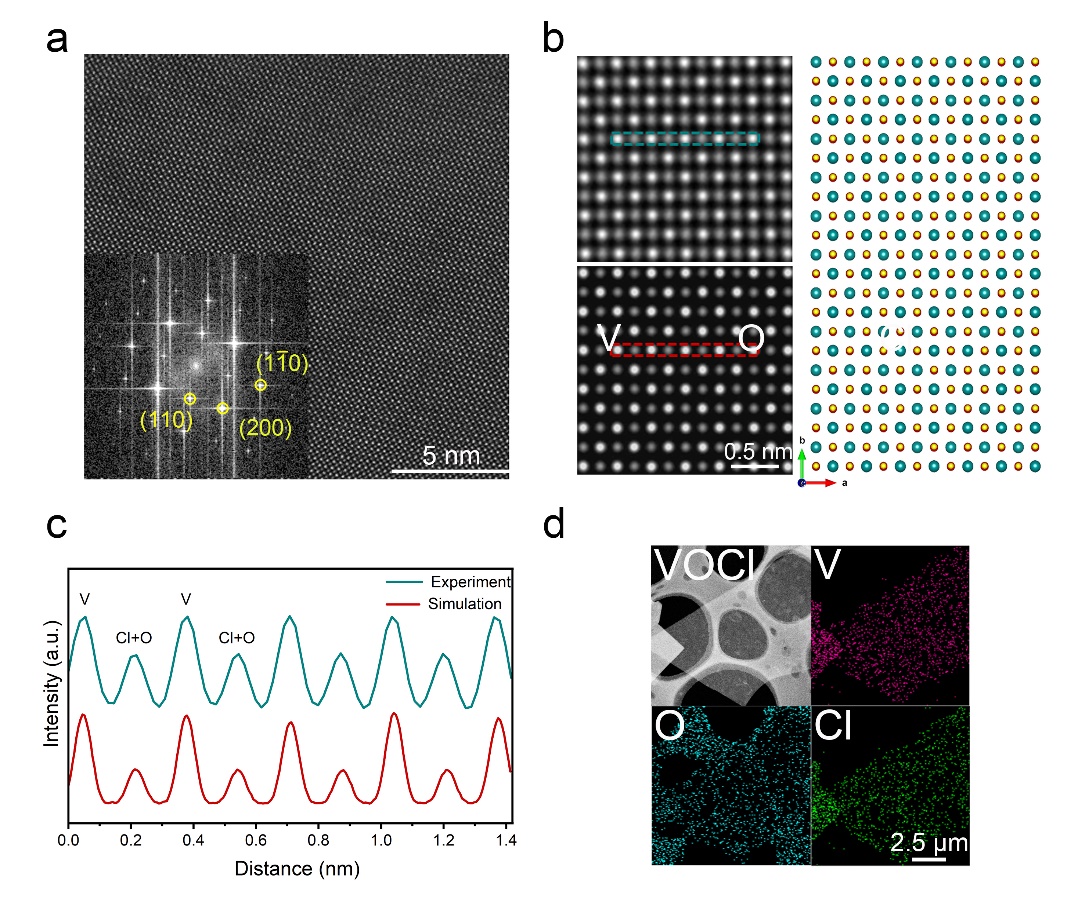


Figure S6. STEM imaging of 2D VOCl.


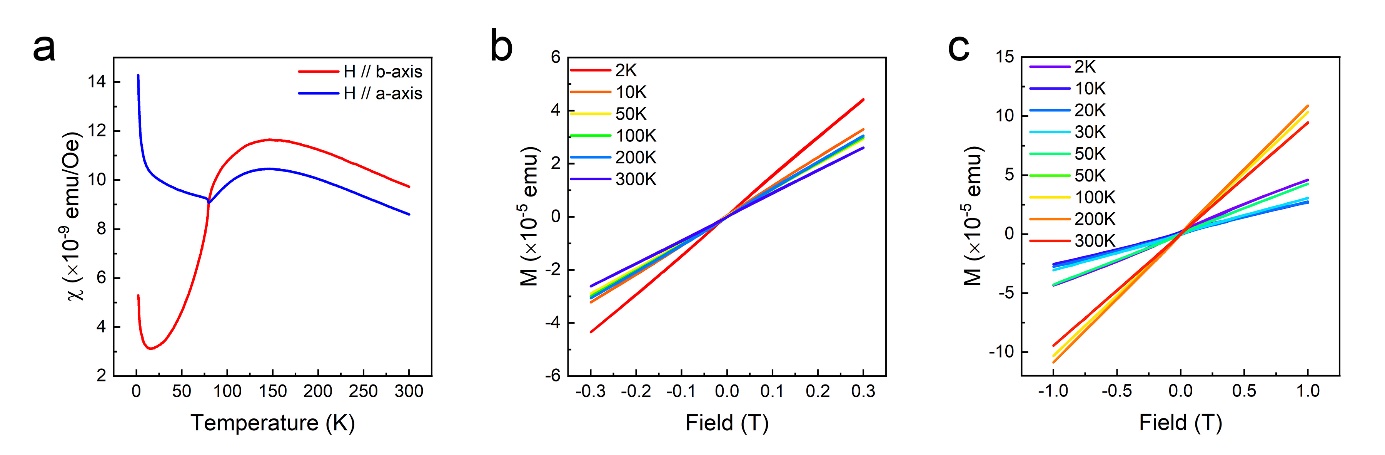


Figure S7. Magnetic characterization for 2D VOCl. a) Temperature-dependent magnetic susceptibility along the *a*-axis and *b*-axis. b and c) M-H curves along *a*-axis and *b*-axis, respectively.


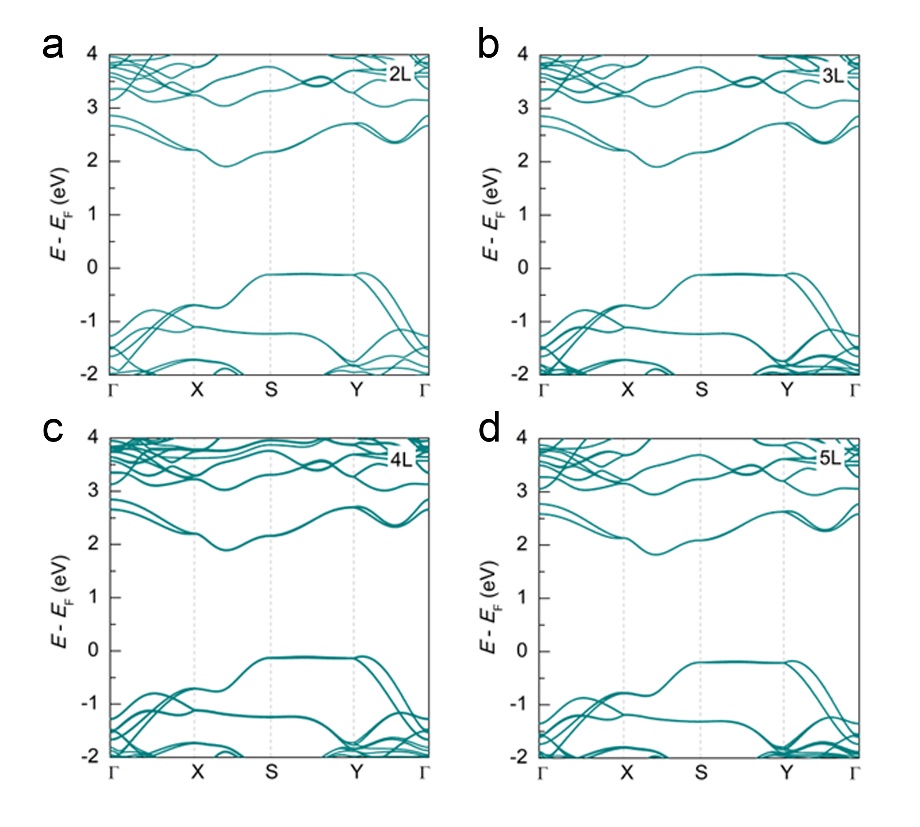


Figure S8. Calculation band structures for 2D VOCl with 2, 3, 4, and 5 layers.


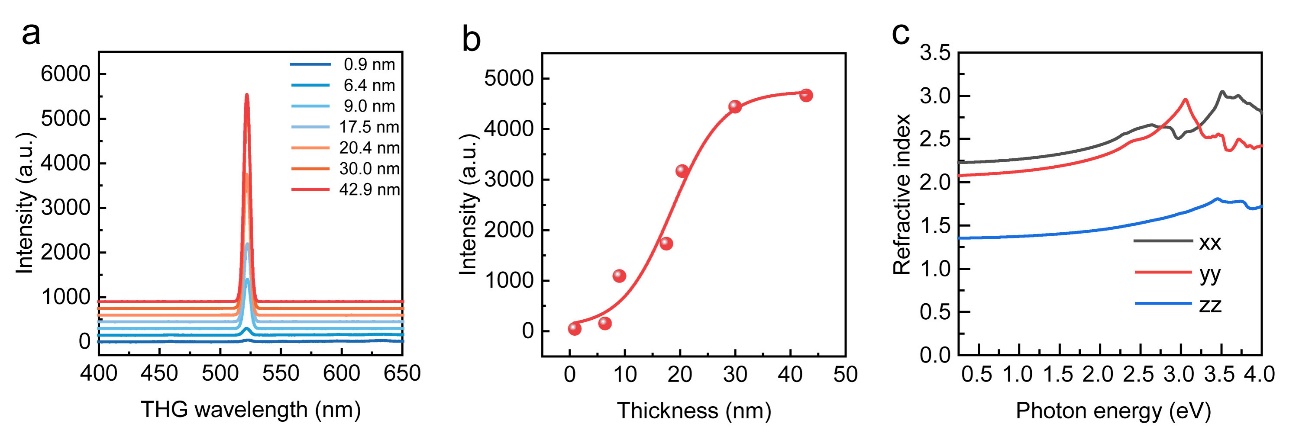


Figure S9. a) Thickness-dependent THG response under excitation at 1558 nm. b) THG intensity variation with respect to thickness of VOCl, extracted from a). c) Calculated refractive index for VOCl crystals.

Figure S9a and b display the thickness-dependent THG intensity for VOCl nanoflakes. The THG intensity increases with the thickness, caused by the longer light-matter interaction length in thicker materials. Before calculating the third-order nonlinear susceptibility (χ^(3)^) of VOCl samples, the coherence length (*l*_coh_) should be considered, where *l*_coh_ = |π/Δ*k*|, and Δ*k* is the phase mismatch. Under the transmission configuration, $\Delta k=k_{3}-3k_{1}=\frac{6\pi\left( n_{1}-n_{3} \right)}{\lambda_{1}}$, where *n*_1_ and *n*_3_ are the refractive indices of the sample at the fundamental (λ_1_) and THG wavelengths (λ_3_), respectively, and *k*_1_ and *k*_3_ are the corresponding wave vectors.^1^ Figure S9c shows that *n*_1_ and *n*_3_ are 2.26 and 2.59 at λ_1_ = 1558 and λ_3_ = 519 nm, respectively. Therefore, *l*_coh_ for VOCl is about 786.9 nm, which is much larger than the samples’ thickness (ranging from 0.9 to 42.9 nm). This indicates that the phase mismatch plays no significant role in the THG intensity for these samples. Then, χ^(3)^ for monolayer VOCl is estimated to be ~ 1.9 × 10^-19^ m^2^/V^2^ by comparing with monolayer WS_2_ (as the reference) using the following equation^2^:

$$\chi_{VOCl}^{(3)}=\frac{d_{WS_{2}}}{d_{VOCl}}\sqrt{\frac{I_{VOCl}}{I_{WS_{2}}}}\chi_{WS_{2}}^{(3)}$$

where $d_{WS_{2}}$ and $d_{VOCl}$ are the thickness of monolayer WS_2_ and VOCl, $I_{WS_{2}}$ and $I_{VOCl}$ are THG intensities of monolayer WS_2_ and VOCl.

VOCl single crystals can be easily exfoliated to nanoflakes due to the weak van der Waals force between interlayers. As shown in Figure S10, the monolayer VOCl is successfully exfoliated onto SiO_2_/Si substrate (the thickness of SiO_2_ film is 285 nm), whose thickness is confirmed by atomic force microscopy (AFM) with the value of ~1.0 nm, consistent with the calculated result of ~0.8 nm. The positions of the three Raman peaks hardly change with increasing the thickness of VOCl nanoflakes, suggesting weak interlayer coupling. No conspicuous Raman peaks are observed in monolayer VOCl, which can be ascribed to the weak phonon vibration modes, poor optical absorption, and interference in VOCl monolayer.


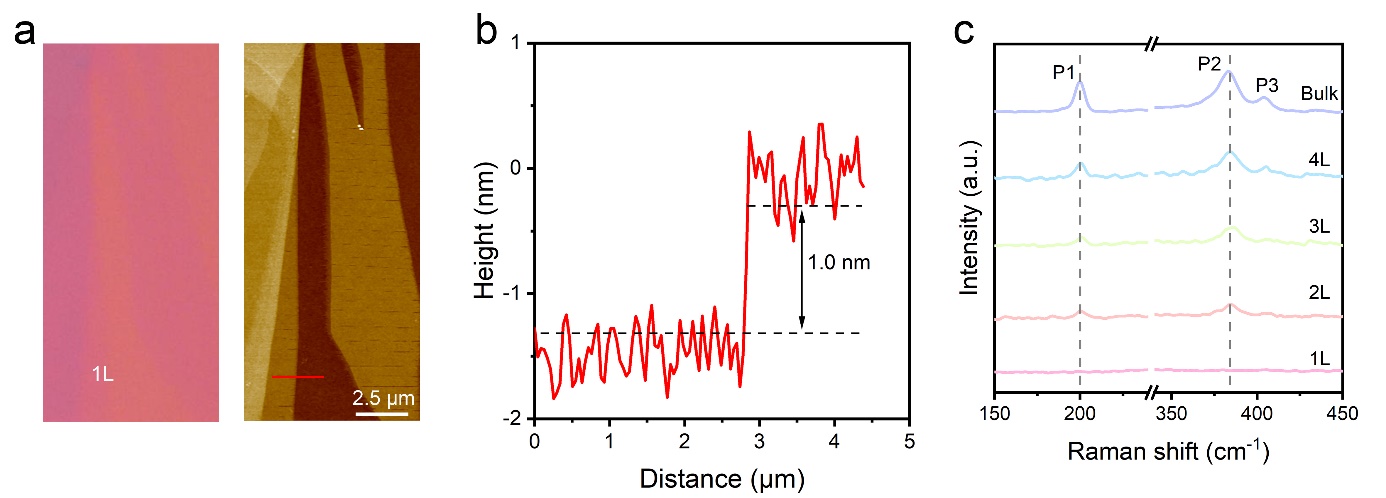


Figure S10. AFM and Raman characterization for VOCl flakes. a) Optical image (left) of exfoliated monolayer VOCl and the corresponding AFM image (right). b) Height profile of the monolayer VOCl along the dashed line in a). The thickness of monolayer VOCl is ~1.0 nm, which is consistent with the calculated monolayer thickness of ~0.8 nm. c) Layer-dependent Raman spectra for VOCl.


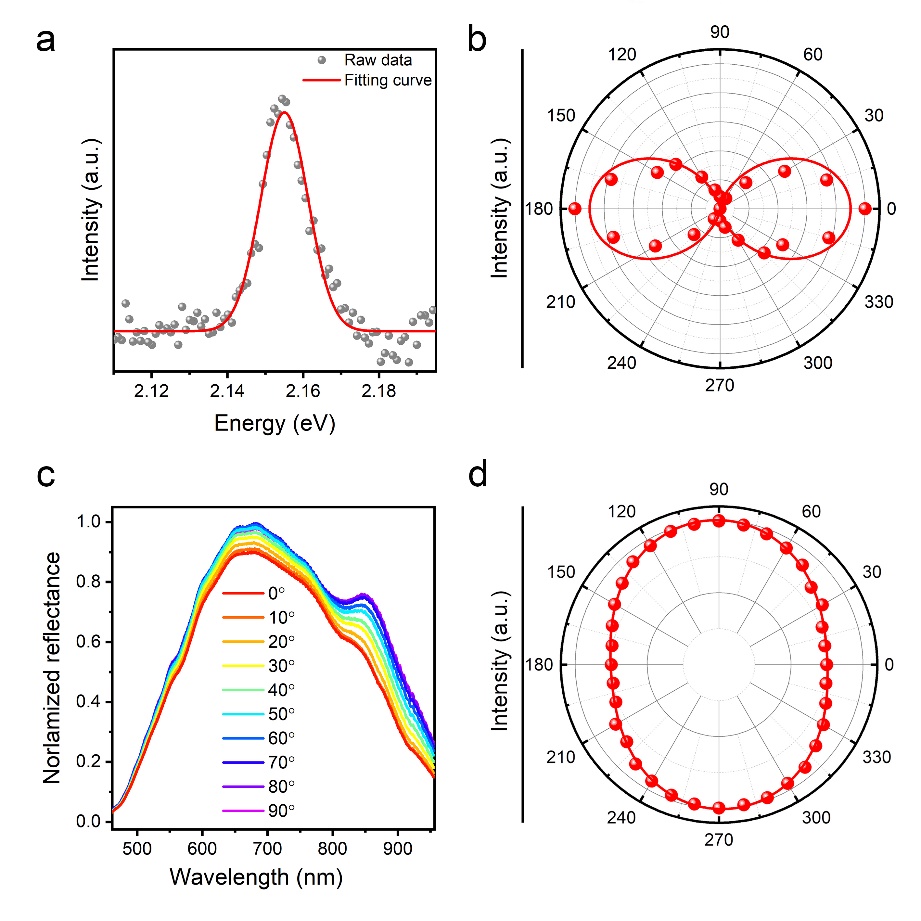


Figure S11. Anisotropic linear optical properties for VOCl nanoflakes. a) Photoluminescence (PL) spectra. b) Polarization-dependent PL intensity under parallel configuration. c) Angle-dependent reflectance spectra in the 450-950 nm range. d) Polarization-dependent reflectance intensity in the wavelength of 850 nm.


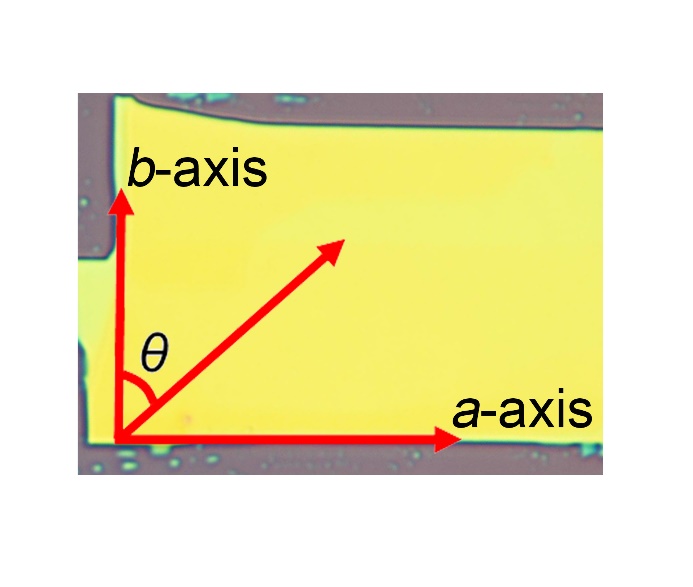


Figure S12. Optical image of VOCl flake.

Raman spectra as a robust characterization method can provide plentiful information for 2D materials, including layer numbers, in-plane anisotropy, phonon modes, and so on. Because VOCl belongs to the orthorhombic system (point group *D*_2h_, mmm). Its Raman active modes are 3*A*_g_ + 3*B*_2g_ + 3*B*_3g_, which all originate from the vibrations of V-Cl bonds. As shown in Figure S13, there are three obvious peaks for bulk VOCl crystals, namely, P1 (~200 cm^-1^), P2 (383 cm^-1^), and P3 (403 cm^-1^), corresponding to *A*_g_ mode. The Raman modes, *B*_2g_ and *B*_3g_, are really weak and can’t be observed due to the negligible electron-photon interactions. Figure S13 displays the ARPRS under the parallel and perpendicular configuration, respectively, which are collected under a 532 nm laser by rotating the crystal with the angle ranging from 0° to 360° (0° is corresponding to the direction along *b*-axis in VOCl crystals). From Figure S13b to d, it can be seen that all Raman peaks show two-fold symmetry, in which the maximum intensities are located at 0° and 180°. In contrast, under perpendicular configuration, all Raman peaks exhibit four-fold symmetry, where the minimum intensities lay at 0°, 90°, 180°, and 270°, respectively. ARPRS demonstrates the in-plane anisotropy of VOCl crystals with *C*_3_ symmetry breaking.


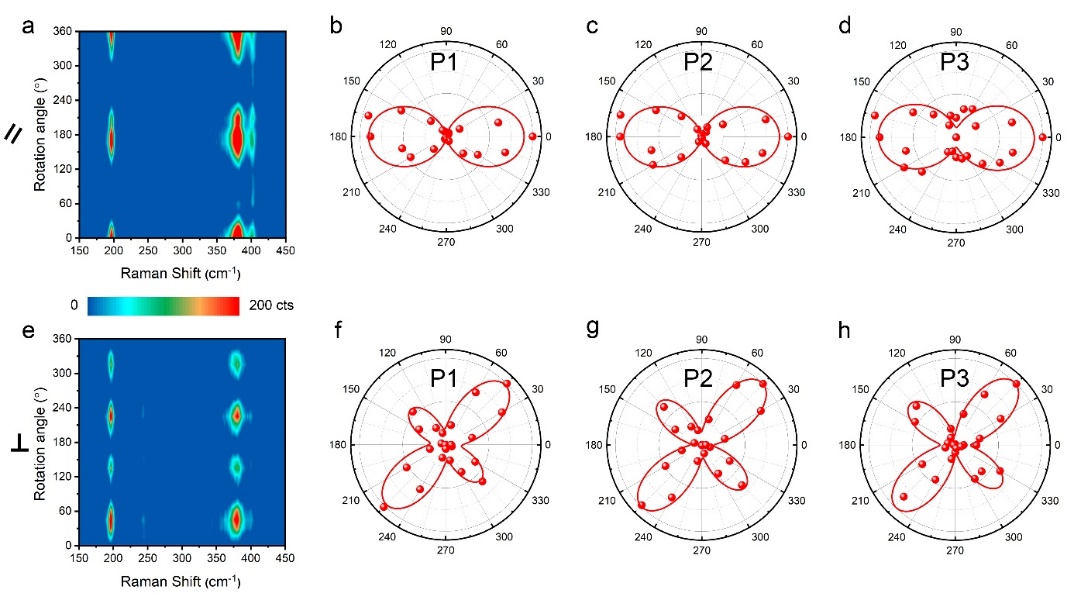


Figure S13. ARPRS for VOCl nanoflakes under the parallel (a-d) and perpendicular configuration (e-h), respectively. Raman peak P1: 200 cm^-1^, P2: 383 cm^-1^, P3: 403 cm^-1^.


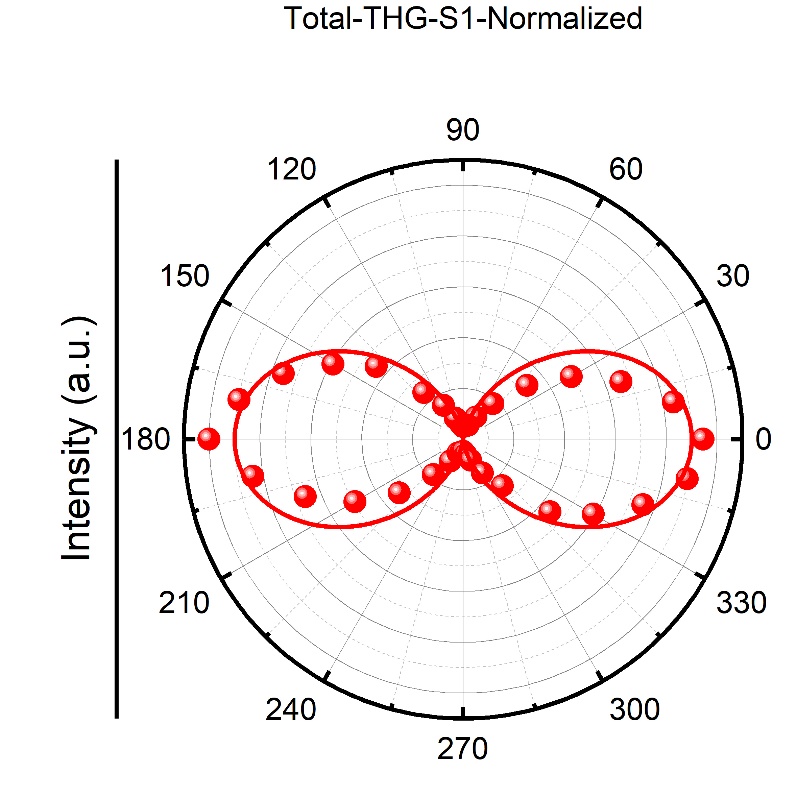


Figure S14. Total THG intensity as a function of polarization angle under the excitation wavelength of 1558 nm.


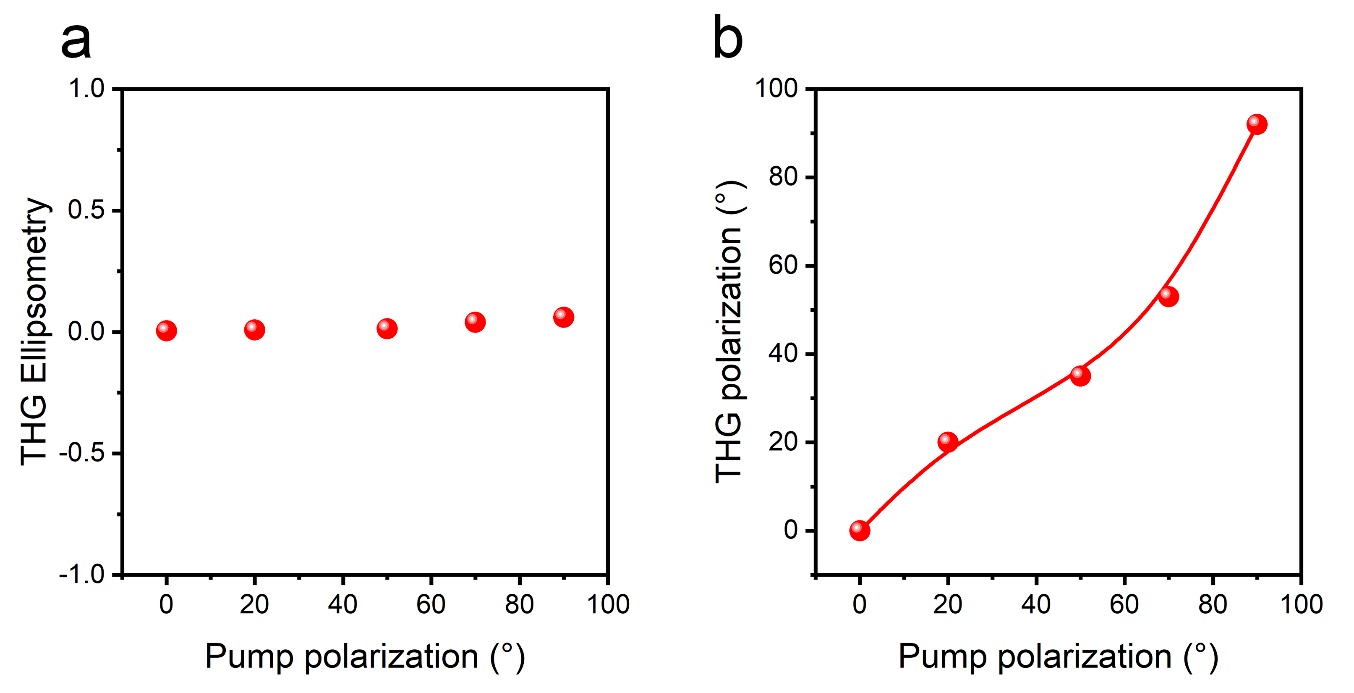


Figure S15. THG ellipsometry (a) and polarization orientation (b) as a function of pump polarization orientation.


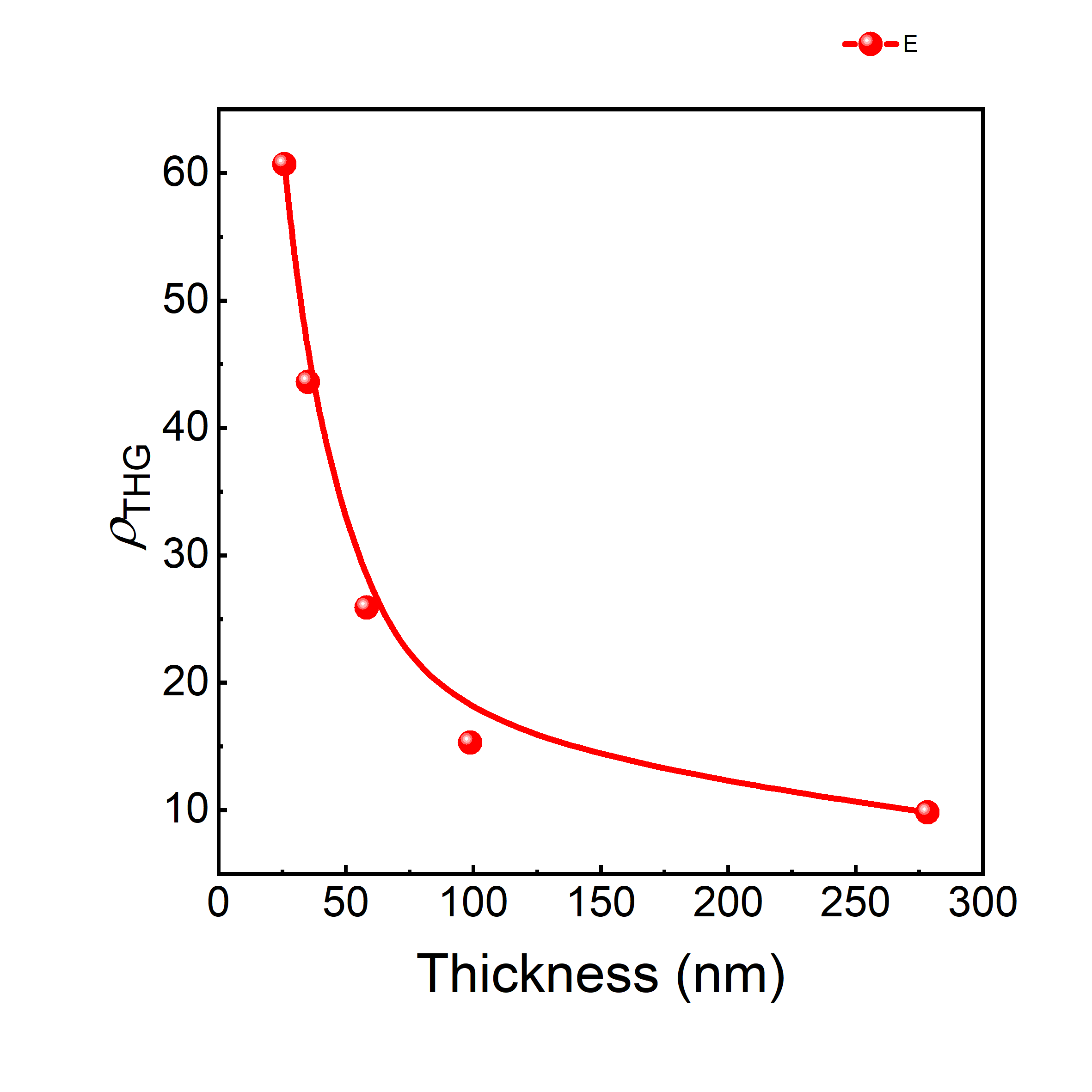


Figure S16. Thickness-dependent THG anisotropic ratio *ρ*_THG_ under 1558 nm excitation wavelength.


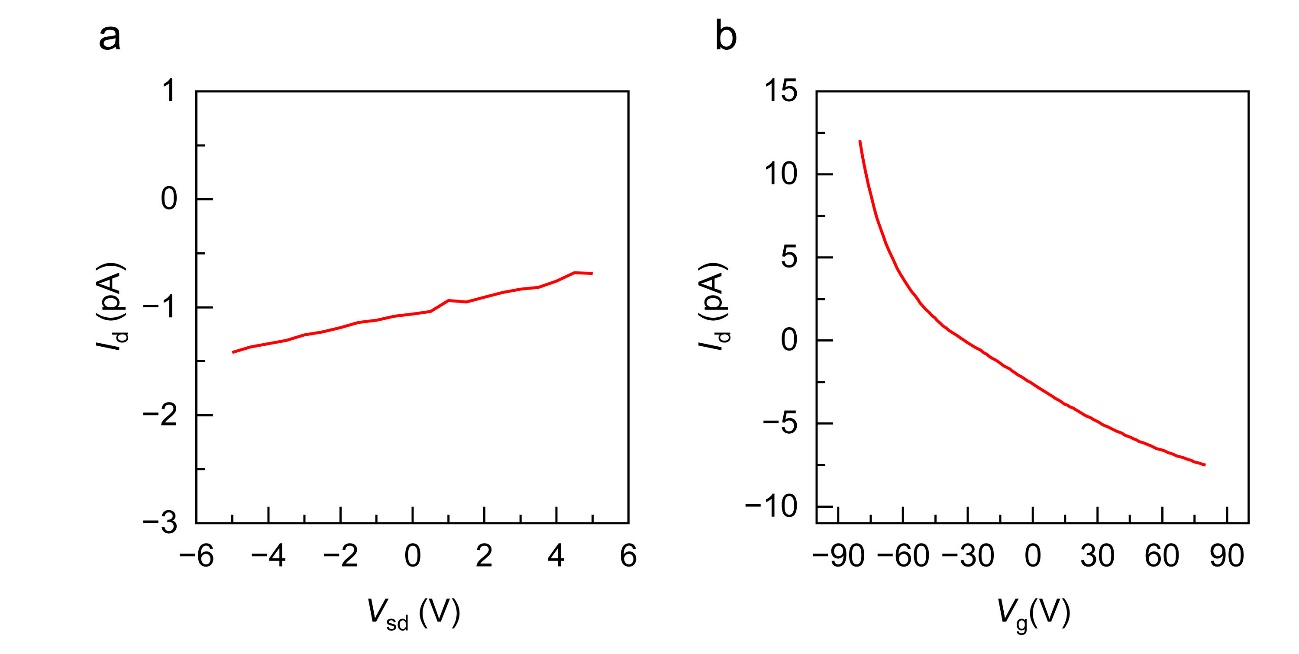


Figure S17. Electrical transport properties of VOCl flakes. a) *I*-*V* curve collected under room temperature. b) Transfer curve at *V*_ds_ = 5 V, which suggests the slight *p*-type conductivity of VOCl flakes.

**Reference**

1 Youngblood, N., Peng, R., Nemilentsau, A., Low, T. & Li, M. Layer-tunable third-harmonic generation in multilayer black phosphorus. *ACS Photonics* **4**, 8-14, doi:10.1021/acsphotonics.6b00639 (2017).

2 Säynätjoki, A. *et al.* Ultra-strong nonlinear optical processes and trigonal warping in MoS_2_ layers. *Nature communications* **8**, 893, doi:10.1038/s41467-017-00749-4 (2017).
